# ﻿The ant genus *Nesomyrmex* Wheeler (Formicidae, Myrmicinae) from the threatened Colombian tropical dry forest: three new species, a new synonymy, and new distributional data

**DOI:** 10.3897/zookeys.1232.141693

**Published:** 2025-03-17

**Authors:** Brandon S. Arredondo, Roberto J. Guerrero

**Affiliations:** 1 Universidad del Magdalena, Santa Marta, Colombia Universidad del Magdalena Santa Marta Colombia

**Keywords:** Biodiversity, conservation, morphology, Neotropical region, taxonomy, threatened ecosystems

## Abstract

The species of the ant genus *Nesomyrmex* inhabiting the tropical dry forest (TDF) of Colombia are reviewed. Three new species of this genus, *Nesomyrmexiku***sp. nov.**, *Nesomyrmexkonina***sp. nov.**, and *Nesomyrmexxerophilus***sp. nov.**, are described based on worker caste. *Nesomyrmexvargasi* Longino, 2006 is recorded for the first time in South America, and *Nesomyrmexantoniensis* (Forel, 1912) is proposed as a junior synonym of *Nesomyrmexasper* (Mayr, 1887). A worker-based taxonomic identification key for the Colombian species is provided. High-resolution images and illustrations, and a distribution map for the species present in the Colombian TDF are provided.

## ﻿Introduction

*Nesomyrmex* Wheeler (Formicidae: Myrmicinae) is a monophyletic genus in the tribe Crematogastrini Forel, 1893 (Ward et al. 2015). These ants are monomorphic and differentiated from any other ant genera of the subfamily Myrmicinae by the combination of the following characters (Bolton 2003; Fernández and Serna 2019): mandibles with 3–5 teeth; median portion of the clypeus with anterior projection, forming a prominent lobe projecting over the dorsum of the mandibles; antenna of 11 or 12 antennomeres with distinct 3-segmented club; rounded or angled propodeal lobe; propodeum armed with a pair of spines of variable lengths.

*Nesomyrmex* ants are small and live mostly on dead or living branches of trees. Currently, 85 species and one subspecies (*Nesomyrmexangulatuslybica* (Menozzi, 1934)) of *Nesomyrmex* are recognized (Bolton 2024) including two extinct species from Dominican amber (De Andrade et al. 1999), with higher species richness concentrated in the tropics and subtropics (Janicki et al. 2016). The genus *Nesomyrmex* occurs in the Afrotropical (30 species; Mbanyana and Robertson 2008; Hita Garcia et al. 2017), Malagasy (32 species; Csősz and Fisher 2015, 2016a, b, c, d), Nearctic (2 species; Smith 1943; Dáttilo et al. 2020), Neotropical (23 species; Kempf 1959; Diniz 1975; Brandão 1991; Bolton 1995; Longino 2006), and Palearctic region (4 species; Collingwood 1985; Collingwood and Agosti 1996; Sharaf et al. 2017, 2020). For Colombia seven species are recorded (Kempf 1959; Fernández et al. 1996; Fernández and Serna 2019), several of which occur in different forest types, including the tropical dry forest (TDF).

The tropical dry forest in Colombia is one of the most threatened terrestrial ecosystems, with a substantial reduction in its extension of approximately 82%, with forest fragments that amount to only 720,000 hectares (Pizano et al. 2014). Agricultural and livestock expansion, the exploitation of non-renewable resources (e.g., mineral coal in the north of the country), urban expansion and tourism are the main factors causing the drastic reduction of TDF in Colombia (Pizano et al. 2016). Despite the ecological importance and imminent systematic reduction, only 5% of the TDF is covered by the national protected areas program, thus increasing concern about the loss of its biodiversity and ecosystem services. Given this panorama, several initiatives have focused on collecting information from different biological models, including ants (Ramos and Guerrero 2023), to develop comprehensive TDF management plans (Pizano et al. 2016).

Since 2020, the research project “Patterns of historical and ecological diversity of ants in the socio-ecosystem of the tropical dry forest of Colombia and its implications for conservation” has been under development. This project integrates multiple approaches such as taxonomy, evolution, and ecology of ants, to understand the conservation status of the TDF in Colombia. In line with this research project, we seek to generate information about the diversity of the different genera of ants in the Colombian TDF. Interestingly, new ant species in the genera *Forelius* (Guerrero and Fernández 2008), *Dorymyrmex* (Cuezzo and Guerrero 2012), and *Pheidole* (Camargo-Vanegas and Guerrero 2020) have been described from the Colombian TDF. Here we review the *Nesomyrmex* ants that inhabit the Colombian TDF, describing three new species, two known so far from the TDF fragments in the Colombian Caribbean. Also, we propose *Nesomyrmexantoniensis* (Forel, 1912) as a junior synonym of *Nesomyrmexasper* (Mayr, 1887), considering the wide morphological variation of the latter’s workers. New distributional data are also provided for each species discussed here, and an illustrated worker-based taxonomic key for the species in Colombia is provided.

## ﻿Materials and methods

### ﻿Specimen processing

**Figure 1.**
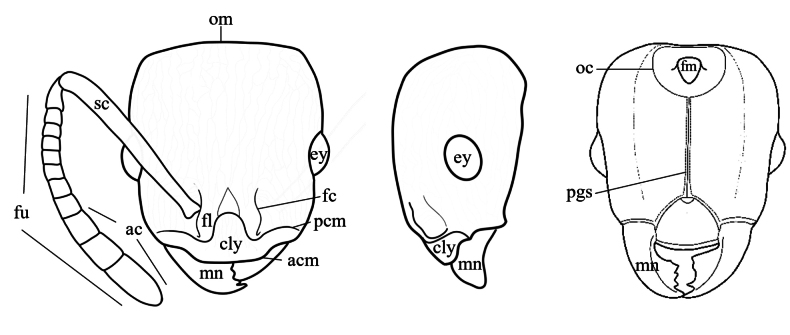
*Nesomyrmex* worker head morphology. Abbreviations: ac (antenal club), acm (anterior clypeal margin), cly (clypeus), ey (eye), fc (frontal carina), fl (frontal lobe), fm (*foramen magnun*), fu (funiculus), gn (gena), mn (mandible), oc (occipital carina), om (occipital margin), pcm (posterior clypeal margin), pgs (postgenal suture), sc (scape).

**Figure 2.**
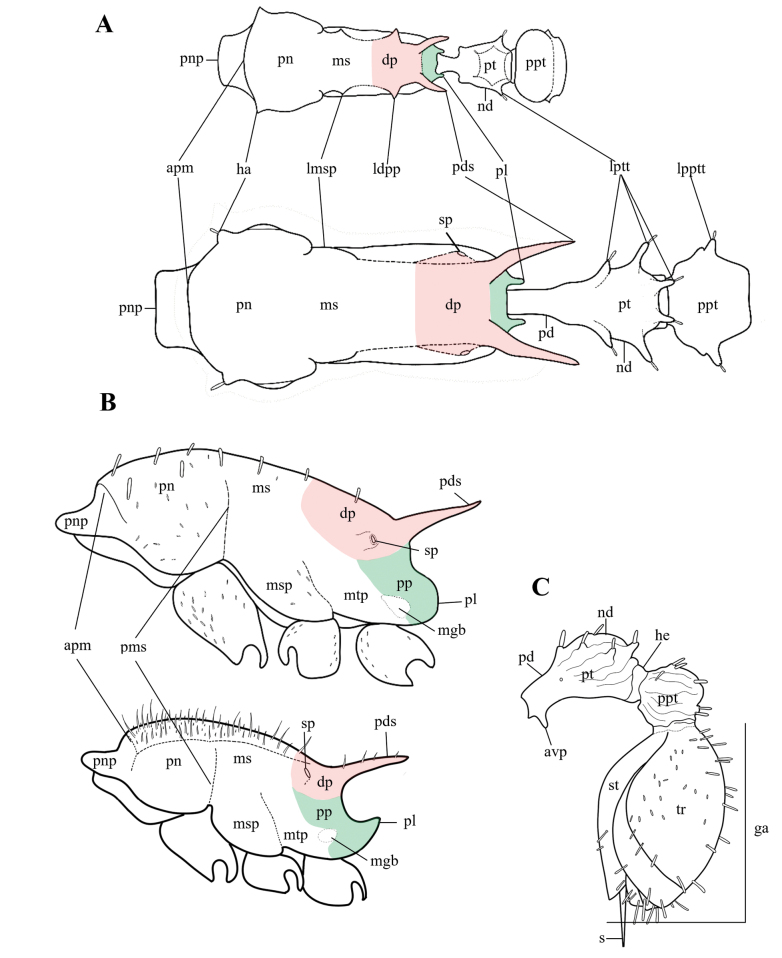
*Nesomyrmex* worker mesosoma and metasoma morphology **A** body in dorsal view **B** mesosoma in lateral view **C** metasoma in lateral view. The red area delimits the dorsopropodeum, while the green area delimits the posteropropodeum. Abbreviations: amp (anterior pronotal margin), avp (anteroventral process), dp (dorsopropodeum), ga (gaster), ha (humeral angles), he (helcium), ldpp (lateral dorsopropodeal process), lmsp (lateral mesonotal process), lpptt (lateral postpetiolar tubercle), lptt (lateral petiolar tubercle), mgb (metapleural gland bulla), ms (mesonotum), msp (mesopleura), mtp (metapleura), nd (node of petiole), pd (peduncle of petiole), pds (propodeal spine), pl (propodeal lobe), pms (promesontal suture), pn (pronotum), pnp (pronotal projection), pp (posteropropodeum), ppt (postpetiole), pt (petiole), sp (spiracle), st (sternite) tr (tergite).

**Figure 3.**
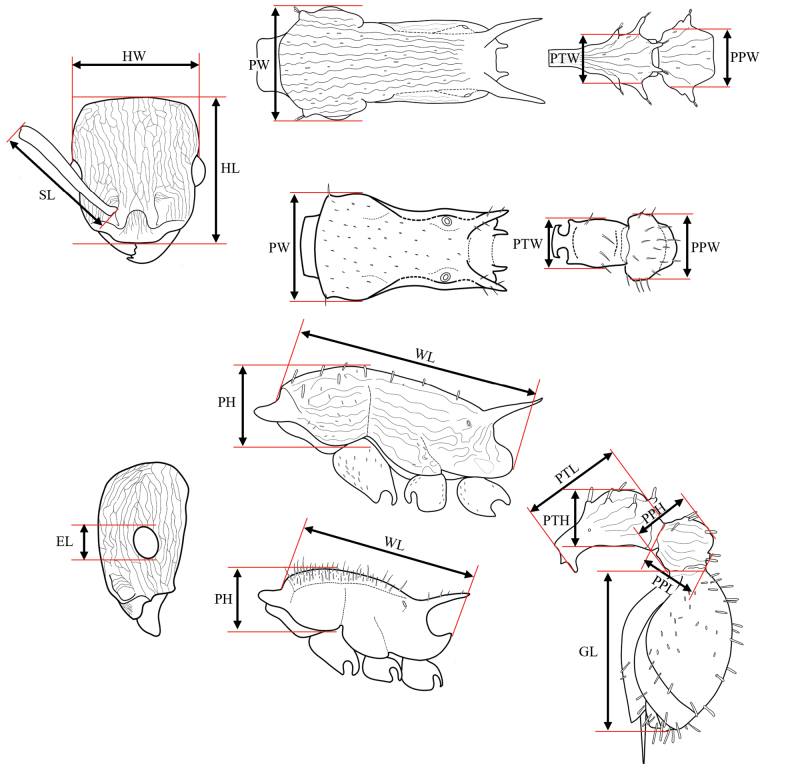
Measurements recorded in the habitus of *Nesomyrmex* worker. Definitions of the acronyms are described in Materials and methods.

Specimens were observed using a Nikon SMZ 745 stereomicroscope. All of them were identified based on taxonomic keys (Kempf 1959), comparison with high-resolution photographs of type material provided by AntWeb (2024) and MZSP. For specimens found that did not correspond to any currently recognized species, the criterion of morphological discontinuity in a comparative framework (Bolton 2000; Longino 2003; Branstetter 2013; Fernández and Serna 2019) was used to support hypotheses of new species. Morphological terminology follows Bolton (2000) and Serna and Mackay (2010). Additionally, head, mesosoma and metasoma regions are illustrated and defined (Figs 1, 2). Sculptural terminology follows Harris (1979). Measurements mainly follow those proposed by Hita Garcia et al. (2017) and Sharaf et al. (2017). Measurements were made with a dual-axis micrometer stage with output in increments of 0.001 mm. However, variation in specimen orientation, alignment of crosshairs with edges of structures, and interpretation of structure boundaries resulted in measurement accuracy to the nearest 0.01 mm. All measurements (Fig. 3) are presented in mm:

**HL** Head length: in full-face view, maximum distance from the midpoint of the anterior margin of the clypeus to the midpoint of the posterior cephalic margin.

**HW** Head width: in full-face view, width of the head immediately posterior to the eyes.

**PW** Pronotal width: in dorsal view, maximum width of pronotum.

**PTW** Petiole width: in dorsal view, maximum width of petiole (if the petiole presents lateral tubercles, this measurement should be taken at the maximum petiole width excluding the tubercles).

**PPW** Postpetiole width: in dorsal view, maximum width of postpetiole (if the postpetiole presents lateral tubercles, this measurement should be taken at the maximum postpetiole width excluding the tubercles).

**PH** Pronotal height: in lateral view, maximum height of the pronotum measured from the lower margin to the upper margin of the pronotum.

**PTH** Petiole height: in lateral view, maximum height of petiolar node.

**PPH** Postpetiole height: in lateral view, maximum height of postpetiole.

**SL** Scape length: in full-face view, maximum length of the scape excluding the neck that occurs just distal of the condylar bulb. Where the scapes were not aligned with the frontal view of the head, it was necessary to place the cephalic capsule obliquely to take a correct measurement of the scape.

**GL** Gaster length: in lateral view, maximum length from the point of insertion with the postpetiole to the most distal part of the gaster, excluding the stinger (this measurement is affected by the degree to which the sternites are retracted or protracted).

**PTL** Petiole length: in lateral view, maximum length of the petiole from the anterior corner of subpetiolar process to the insertion with the postpetiole.

**PPL** Postpetiole length: in lateral view, maximum length from the most anterior point of insertion with the petiole to the insertion of the gaster.

**WL** Weber’s length: in lateral view, diagonal length of the mesosoma from the most anterior point of the pronotal slope (excluding the pronotal projection), to the posterior margin of the propodeal lobe.

**EL** Eye length: in lateral view, maximum diameter of the compound eye.

**Indices**

**CI** Cephalic index: (HW/HL) *100.

**SI** Scape index: (SL/HW) *100.

### ﻿Specimen drawing, imaging, and distribution maps

The drawings of the different *Nesomyrmex* species recorded here were created using Sketchbook Pro v. 9.0. Stacked color images of the species were generated at Laboratorio de equipos ópticos del Departamento de Biología (Universidad Nacional de Colombia, Sede Bogotá) and Laboratorio de Entomología de la Universidad de la Amazonia (LEUA), using a Leica M205A Auto-Montage with integrated camera (DFC450) and LAS v. 4.6 software. Images were edited and organized (Adobe Photoshop v. 25.3.1) to improve image characteristics such as brightness and contrast. The species distribution maps were made using the information obtained from the different labels of the specimens; this information was processed with the Quantum Gis v. 3.36 software (QGIS 2024).

### ﻿Repositories

We examined specimens deposited in the following collections. Those marked with an asterisk (*) are not registered in Evenhuis (2024).

**CASC**California Academy of Sciences, USA

**CBUMAG**Centro de Colecciones Biológicas de la Universidad del Magdalena, Santa Marta, Magdalena, Colombia

**CTNI** Colección Taxonómica Nacional de Insectos Luis María Murillo, Corporación Colombiana de Investigación Agropecuaria-AGROSAVIA, Tibaitatá, Mosquera, Cundinamarca, Colombia. CELM in Evenhuis (2024)

**IAvH** Colección de Entomología del Instituto de Investigaciones de recursos biológicos Alexander von Humboldt, Villa de Leyva, Boyacá, Colombia

**ICN** Colección de Entomología del Instituto de Ciencias Naturales, Universidad Nacional de Colombia, Bogotá D. C., Colombia

**MEFLG**Museo Entomológico Francisco Luis Gallego, Universidad Nacional de Colombia, Medellín. Medellín, Antioquia, Colombia

**MHNG** Muséum d’Histoire Naturelle, Geneva, Switzerland

**MPUJ**Colección de Artrópodos del Museo de Historia Natural de la Pontificia Universidad Javeriana. Bogotá D. C., Colombia

**MSNG**Museo di Storia Naturale Giacomo Doria, Genova, Italy

**MUSENUV**Museo de Entomología de la Universidad del Valle, Valle del Cauca, Santiago de Cali, Colombia

**MZSP**Museo de Zoologia da Universidade de São Paulo, São Paulo, Brazil

**NHMW**Naturhistorisches Museum, Vienna, Austria

**UCDC**R.M. Bohart Museum of Entomology, Davis, CA, USA

**UNAB**Museo Entomológico Universidad Nacional de Colombia, Facultad de Ciencias Agrarias, Bogotá. D. C., Colombia

## ﻿Results

### ﻿Synoptic list of *Nesomyrmex* species from the tropical dry forest in Colombia

*Nesomyrmexasper* (Mayr, 1887)

= *Nesomyrmexantoniensis* (Forel, 1912), syn. nov.

*Nesomyrmexechinatinodis* (Forel, 1886)

*Nesomyrmexiku* sp. nov.

*Nesomyrmexkonina* sp. nov.

*Nesomyrmexpittieri* (Forel, 1899)

*Nesomyrmexpleuriticus* (Kempf, 1959)

*Nesomyrmexspininodis* (Mayr, 1887)

*Nesomyrmexvargasi* Longino, 2006

*Nesomyrmexxerophilus* sp. nov.

### ﻿Key to Colombian *Nesomyrmex* species based on workers

(adapted and modified from Kempf (1959), * species probably present in Colombia)

**Table:** 

1	Antenna with 12 antennomeres	**2**
–	Antenna with 11 antennomeres	**9**
2	Antennal scape in repose reaching or surpassing the occipital margin (SI > 82) (Fig. 4A). In dorsal view, humerus (ha) distinctly dentate (Fig. 5A). Mesosoma length (= WL) greater than or equal 1.4 mm	**3**
–	Antennal scape in repose failing to reach the occipital margin (SI < 81) (Fig. 4B). In dorsal view, humerus (ha) distinctly angled (Fig. 5B), Slightly angled (Fig. 5C) or rounded (Fig. 5D). Mesosoma length (= WL) less than or equal 1.3 mm	**4**
3	In dorsal view, lateral mesonotum process (lmsp) dentate (Fig. 6A). Lateral margins of the mesosoma constricted anteriorly and posteriorly to the mesonotal projection	*N.pulcher* (Emery)*
–	In dorsal view, mesonotum lacking lateral projections, sometimes rounded or slightly rounded margins (Fig. 6B). Lateral margins of mesonotum weakly constricted anteriorly and posteriorly to mesonotum	***N.anduzei* (Weber)**
4	Dorsal surface of the head longitudinally costate (Fig. 7A) or rugose (Fig. 7B)	**5**
–	Dorsal surface of the head foveate (Fig. 7C), or smooth and shiny (Fig. 7D)	**7**
5	Frontal lobe projected laterally (widest posterior to torulus) (Fig. 8A), covering the antennal insertions. In dorsal view, lateral projection of the mesonotum dentate (lmsp) (Fig. 9A). Lateral dorsopropodeal processes (ldpp) long, covering propodeal spiracle in dorsal view (Fig. 9A). Dorsal surface of mesosoma and postpetiole with longitudinal costae strongly elevated	***N.xerophilus* sp. nov.**
–	Frontal lobes without lateral projection, antennal insertions partially exposed (Fig. 8B). In dorsal view, lateral projection of the mesonotum blunt (lmsp) (Fig. 9B). Lateral dorsopropodeal processes (ldpp) short, not covering spiracle in dorsal view (Fig. 9B). Dorsal surface of mesosoma and postpetiole with weakly elevated longitudinal roughness	**6**
6	In lateral view, mesosomal profile straight (Fig. 10A). In dorsal view, humerus (ha) strongly angled (Fig. 5B)	***N.iku* sp. nov.**
–	In lateral view, mesosomal profile convex (Fig. 10B). In dorsal view, humerus (ha) slightly angulated (Fig. 5C)	***N.itinerans* (Kempf)**
7	Dorsal surface of the head smooth and shiny (Fig. 7D). Head length > 1.1 ×mesosoma length. Anterior margin of pronotum without carina (Fig. 11A). Postpetiolar node smooth and shiny	***N.tonsuratus* (Kempf)**
–	Dorsal surface of the head foveate, or weakly foveated (Fig. 7C). Head length smaller than mesosoma length. Anterior margin of pronotum carinate (Fig. 11B). Postpetiolar node sculpted and matte	**8**
8	Dorsal surface of the head foveate. Antennal scape curved at its base. Propodeal spines as long as the distance between their apices	***N.pittieri* (Forel)**
–	Dorsal surface of the head weakly foveated. Antennal scape straight at its base. Propodeal spines short and subconical, much shorter than the distance between their apices	***N.brasiliensis* (Kempf)** *
9	Antennal scape in repose reaching or surpassing the occipital margin (S > 82) (Fig. 4A). Propodeal lobe (pl) angulate, apex blunt (Fig. 12A)	**10**
–	Antennal scape barely reaching the occipital margin by a distance equal to its greatest width (SI < 81) (Fig. 4B). Propodeal lobe (pl) short and uniformly rounded (Fig. 12B)	**13**
10	Inner area of the dorsal surface of the mandibles finely imbricate and subopaque (Fig. 13A). Sides of the mesosoma roughly sculptured	***N.asper* (Mayr)**
–	Inner area of the dorsal surface of the mandible smooth and shiny (Fig. 13B). Sides of the mesosoma smooth, with little or no roughness	**11**
11	Dorsal surface of the head rugose (Fig. 7B), matte sculpture. Legs lacking erect hairs (Fig. 14A)	*N.brimodus* (Bolton)*
–	Dorsal surface of the head smooth and shiny (Fig. 7D). Legs with long erect hairs (Fig. 14B)	**12**
12	Anterior margin of pronotum straight (Fig. 15A). Sculpture of mesosoma dorsum opaque and strongly marked, with reticulate microsculpture. Sides of mesosoma with variable sculpture, but never longitudinally striate	***N.pleuriticus* (Kempf)**
–	Anterior margin of pronotum convex (Fig. 15B). Sculpture of dorsum of mesosoma partially smooth and shiny, with weakly marked longitudinal striae. Sides of mesosoma with longitudinal striations	***N.vargasi* Longino**
13	Dorsal surface of the head smooth and shiny. Mesosoma with weakly marked longitudinal striation. First tergite gastral without sculpture, completely smooth and shining (Fig. 16A). Coloration uniformly pale yellow	***N.konina* sp. nov.**
–	Dorsal surface of the head with sculpture, partly or completely rugose. Mesosoma with marked longitudinal striation. First gastral tergite with variable sculpture (Fig. 16B). Testaceous or yellowish brown to dark brown coloration	**14**
14	In full-face view, front and vertex opaque, finely reticule-punctate and longitudinally rugose, without shining areas (Fig. 17A). Coloration concolor, testaceous or yellowish-brown. First gastric tergite usually more sharply aciculate-striate on the anterior half gastric tergite	***N.spininodis* (Mayr)**
–	In full-face view, front and vertex partly or completely smooth and shiny (Fig. 17B). Color always darker, at least head and gaster brown to black. First gastric tergite very lightly sculptured	***N.echinatinodis* (Forel)**

**Figure 4.**
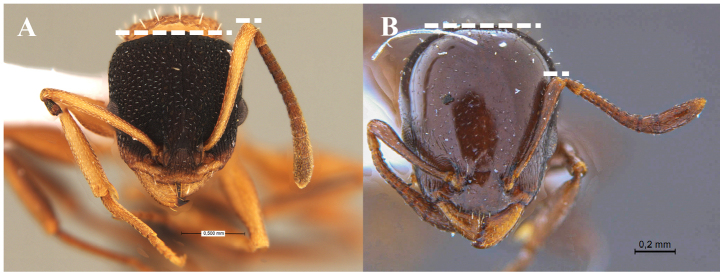
Relative length of antennal scapes **A** scape in repose reaching or surpassing the occipital margin **B** scape in repose failing to reach the occipital margin.

**Figure 5.**
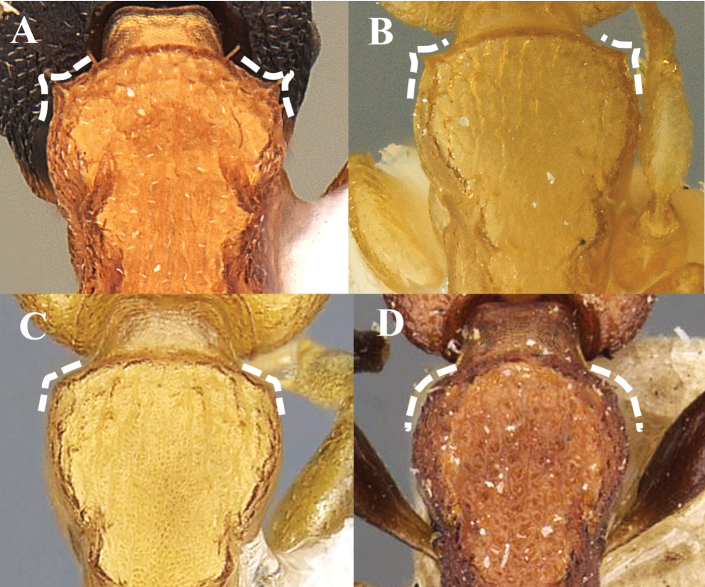
Humerus shape (dorsal view) **A** dentate **B** strongly angled **C** slightly angled **D** rounded.

**Figure 6.**
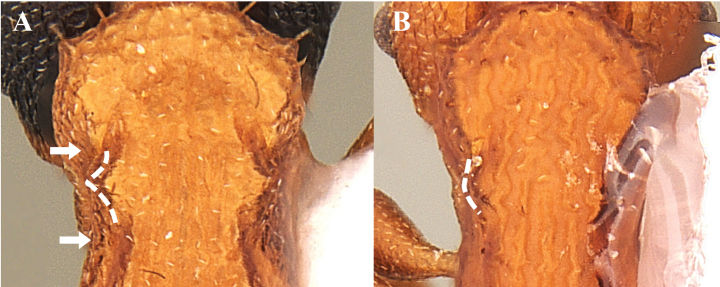
Mesonotal lateral projection **A** triangular lobe-shaped **B** rounded or slightly rounded lobe-shaped. White arrows indicate anterior and posterior constrictions to the lateral projection of the mesonotum.

**Figure 7.**
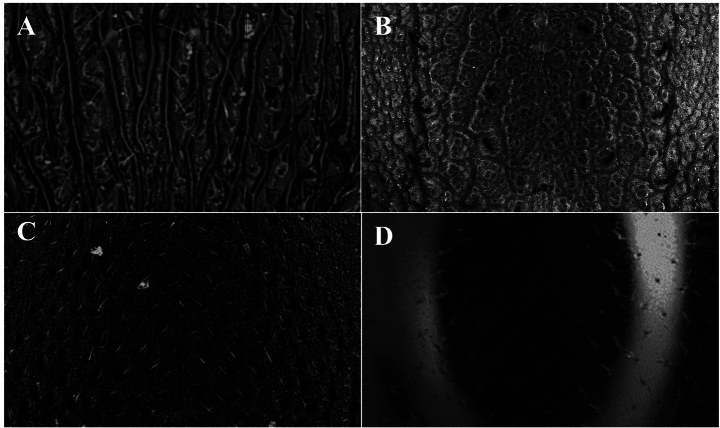
Types of sculpture **A** costate **B** rugose **C** foveate **D** smooth.

**Figure 8.**
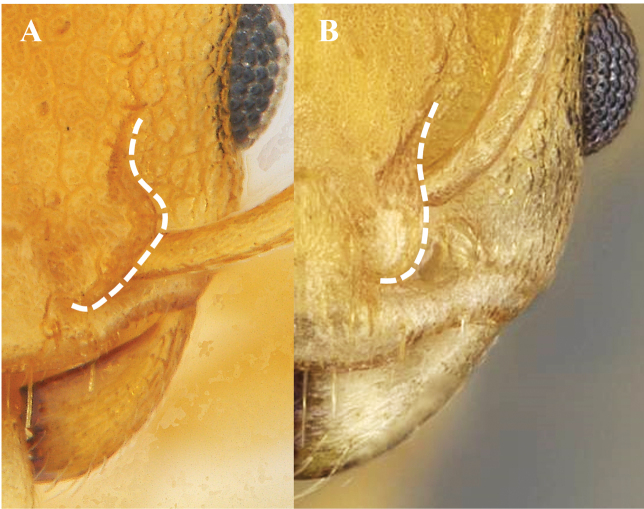
Frontal lobes **A** with lateral expansion **B** without lateral projection.

**Figure 9.**
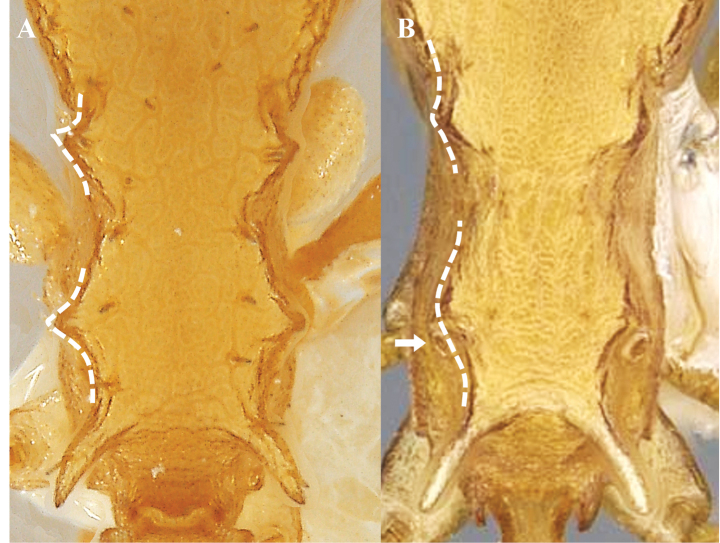
Mesosomal dorsum, in dorsal view **A** lateral projection of the mesonotum dentate, dorsopropodeum projected laterally over the spiracle **B** lateral projection of the mesonotum angled, dorsopropodeum without lateral projection over the spiracle. White arrows indicate the propodeal spiracle.

**Figure 10.**
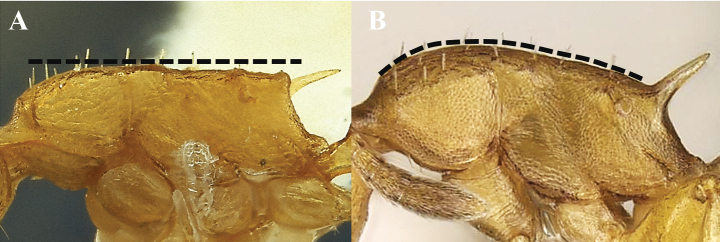
Mesosomal dorsal profile **A** straight **B** convex.

**Figure 11.**
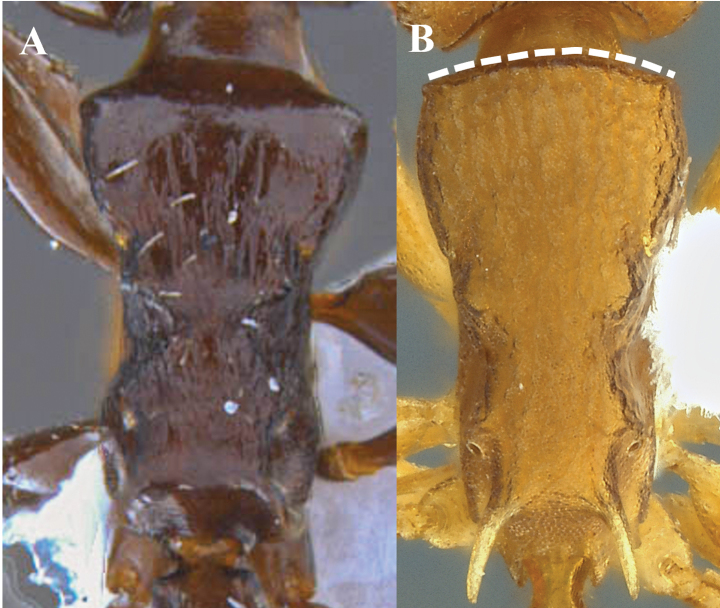
Anterior margin of pronotum **A** without carina **B** carinate.

**Figure 12.**
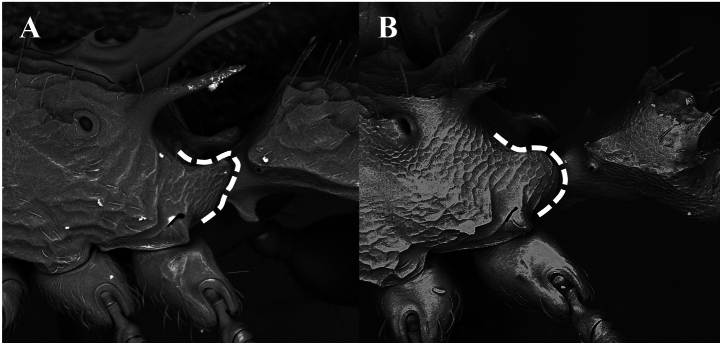
Propodeal lobe shape **A** angulate, apex blunt **B** short, uniformly rounded.

**Figure 13.**
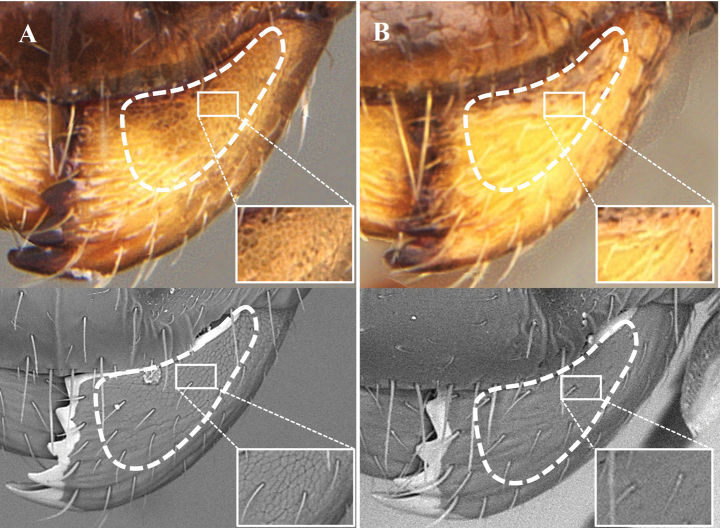
Medial area of the dorsal surface of the mandible, photomicrography and electron micrography **A** reticulated and opaque **B** smooth and shiny. White box is a close-up to observe the detail of the sculpture in question.

**Figure 14.**
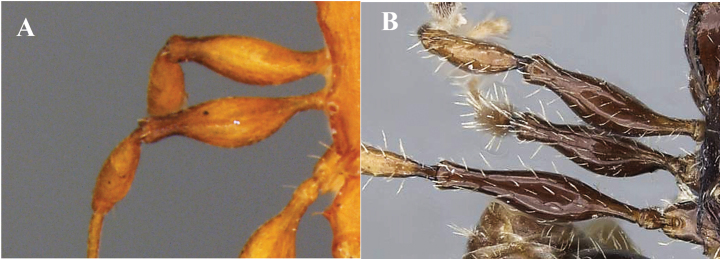
Leg pilosity **A** without presence of pilosity **B** with long erect or suberect hairs.

**Figure 15.**
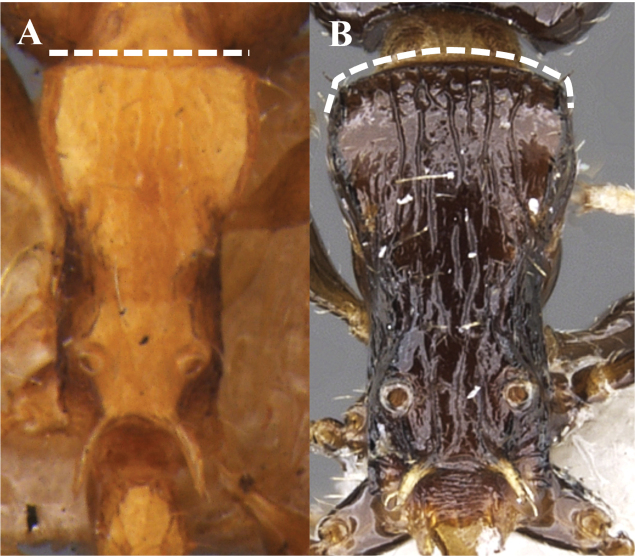
Anterior margin of pronotum **A** straight **B** convex.

**Figure 16.**
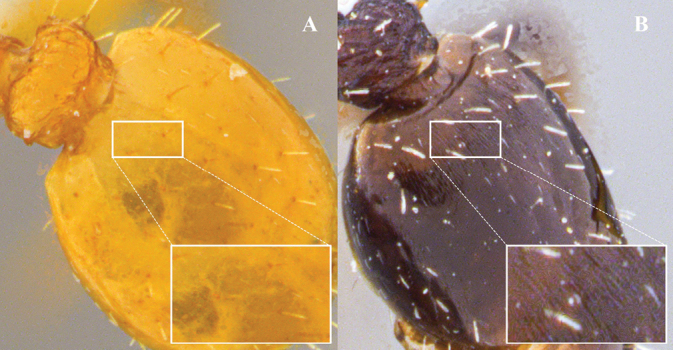
Sculpting of the first gastral tergite **A** smooth **B** sculpted. The inset square corresponds to an enlargement of the dorsal surface of the first gastral tergum.

**Figure 17.**
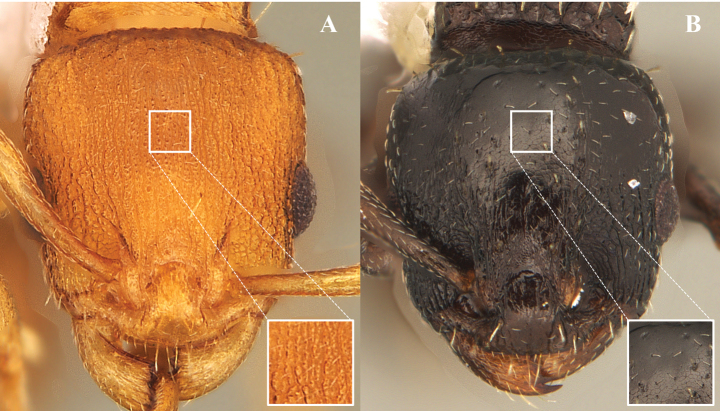
Sculpting of front and vertex (full-face view) **A** finely reticulate-punctate and longitudinally rugose, without shining areas **B** partly or completely smooth and shining. White box is a close-up to observe the detail of the sculpture in question.

### ﻿Species accounts

#### ﻿ Nesomyrmex asper

Taxon classificationAnimaliaHymenopteraFormicidae

(Mayr, 1887)

3DBF20F9-0103-5E86-9B89-A4EE102E7899

**Figure 18.**
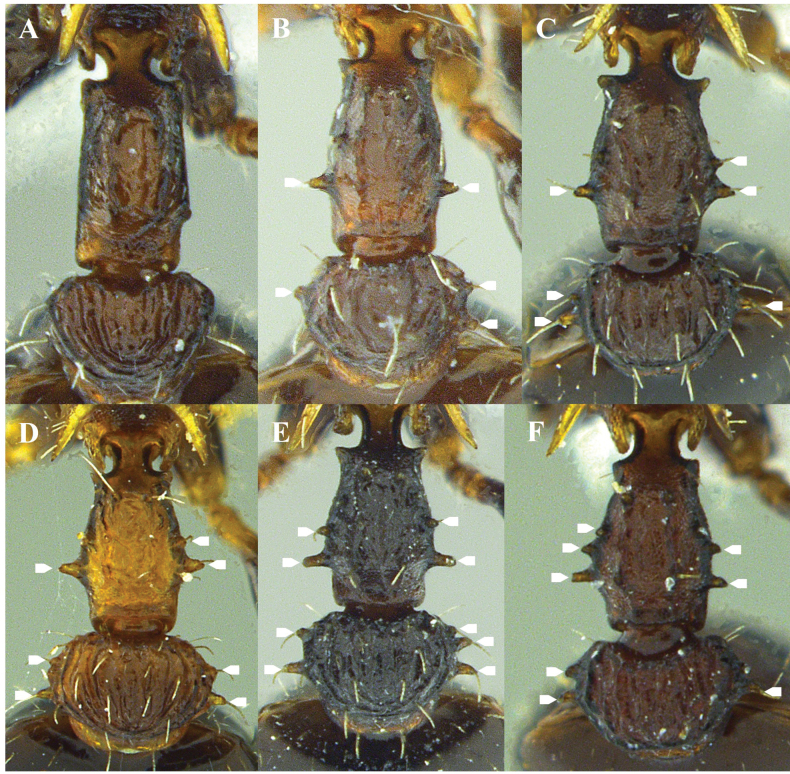
Variation of lateral petiole tubercles in *Nesomyrmexasper*. White arrows indicate the tubercles.

**Figure 19.**
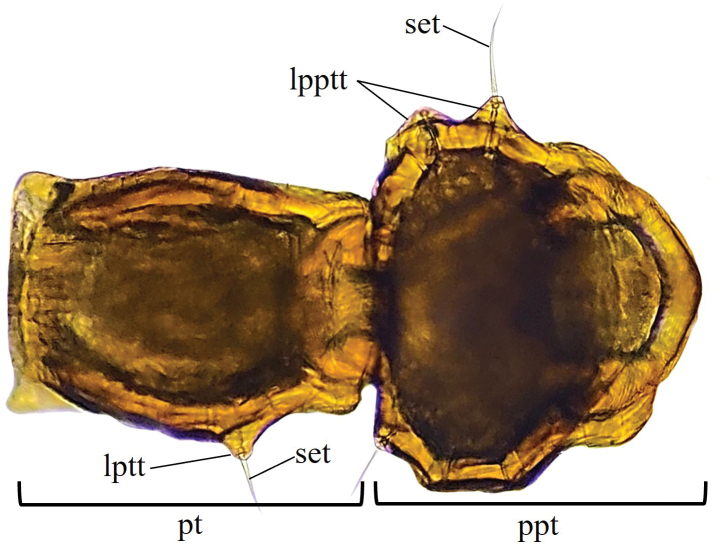
Lateral petiole and postpetiole tubercles (Setigerous tubercles). Abbreviations: lpptt (lateral postpetiolar tubercles), lptt (lateral petiolar tubercles), pt (petiole), ppt (postpetiole), set (seta).

**Figure 20.**
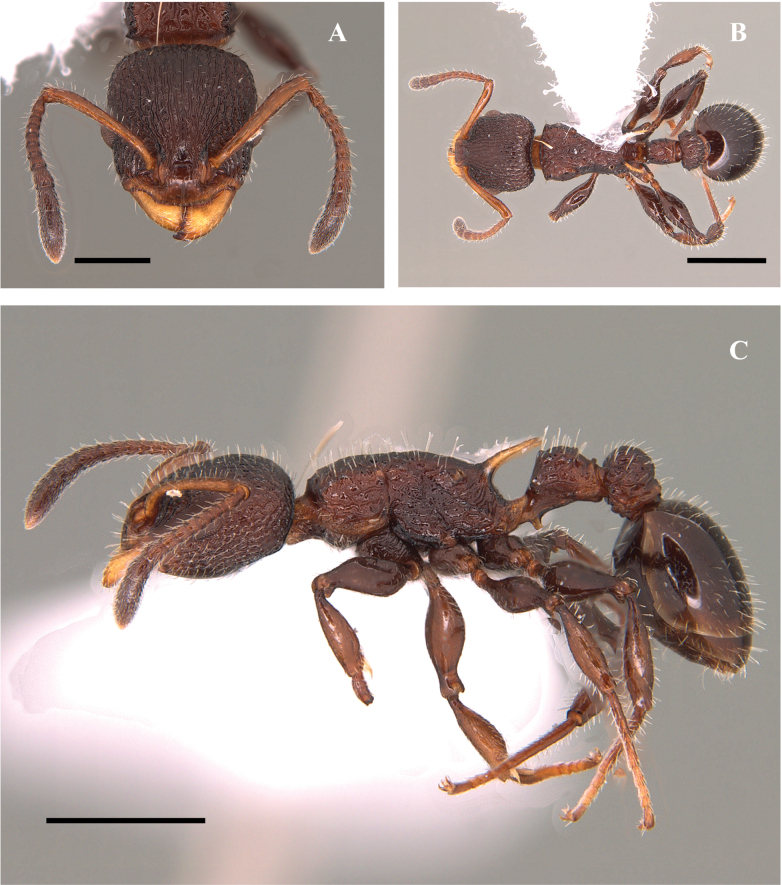
*Nesomyrmexasper* worker (ICN 106553) **A** full-face view **B** lateral view **C** dorsal view. Scale bars: 0.5 mm (**A**); 1.0 mm (**B**).

Figs 18 , 19 , 20 , 29

Leptothorax asper Mayr, 1887: 618. Syntype series (worker, queen, male): Brazil, Santa Catarina. [NHMW] (AntWeb image examined, CASENT0901801).
Combination in Leptothorax (Goniothorax): Emery 1896: 59.
Leptothorax (Goniothorax) asper var. rufa Emery, 1896: 61. Syntype series (queen, worker): Brazil, Pará. [MSNG]. (AntWeb image examined, CASENT0904722). Synonymy by Kempf 1959: 414.
Leptothorax (Goniothorax) tristani Emery, 1896: 61. Syntype series (queen, worker): Costa Rica. [MSNG]. (AntWeb image examined, CASENT0904724). Synonymy by Longino 2006: 136.
Leptothorax asper var. antoniensis Forel, 1912: 18. Syntype series. (worker): Colombia, Sierra Nevada de Santa Marta, San Antonio. [MHNG]. (AntWeb image examined, CASENT0909002). syn. nov.
Leptothorax asper var. sulfurea Forel, 1912: 18. Syntype series (worker): Brazil. [MHNG]. (AntWeb image examined, CASENT0909003). Synonymy by Kempf (1959: 414).
Combination in Leptothorax (Nesomyrmex): Kempf 1959: 414.
Combination in Nesomyrmex: Bolton 2003: 272.

##### Worker measurements.

(*n* = 8) HL 0.86–1.08, HW 0.78–0.99, SL 0.68–0.84, ML 0.41–0.53, EL 0.19–0.24, PW 0.51–0.66, PTW 0.21–0.31, PPW 0.31–0.44, WL 1.05–1.38, PH 0.29–0.34, PTL 0.37–0.46, PTH 0.23–0.29, PPL 0.24–0.34, PPH 0.26–0.32, GL 0.88–1.01. CI 90–92, SI 84–87.

##### Geographic range.

Argentina, Bolivia, Colombia, Ecuador, French Guaina, Nicaragua, Panama, Paraguay, Trinidad and Tobago, Venezuela

##### Examined material.

Colombia • 1 worker; Atlántico, Usicurí, Vda. Luriza, CIALU; 10.75198°N, 75,03075°W; 155 m a.s.l.; 28–30 Mar. 2023; J. Camargo, H. Sierra, S. De la Hoz legs.; Winkler; CBUMAG:ENT:54657 • 1 worker; Bolívar, Turbaco, Finca el Huerto; 10.371944°N, 75.349667°W; 102 m a.s.l.; 05 Jul. 2015; A. Sagoval, C. Cantor legs.; secondary forest; ICN 106553. • 1 worker; Cesar, La Jagua de Ibirico; 9.561111°N, 73.336389°W; 150 m a.s.l.; 2007; F. Fernández leg.; pitfall; ICN 019802. • 1 worker; Huila, Aipe, Cuenca Río Aipe, Vda. San Isidro; 3.3453561°N, 75.3057781°W; 841 m a.s.l.; 25 Nov. 2017; L. Arcila leg.; Winkler; IAvH. • 1 worker; Valle del Cauca, Dagua, Cuenca Río Dagua, Vda. Limonar; 3.6238689°N, 75.6945069°W; 1071 m a.s.l.; 17 Aug. 2021; L. Arcila leg.; Pitfall; IAvH. • 2 workers; Cauca, Santander de Quilichao, Las Chatas; 3.1096°N, 76.5265°W; 909 m a.s.l.; 17 Aug. 2021; M. A. Bautista-Giraldo; I. Armbrecht legs.; meadow; CBUMAG:ENT:40121, CBUMAG:ENT:40122.

##### Natural history.

Some workers were collected foraging during the day on vegetation in TDF fragments in the Valle del Cauca. Additionally, one worker was collected foraging on the grassland soil using pitfall traps.

##### Comments.

This species is recognized by the set of the following characters: 11 antennomeres, clypeus sculpture ranges from smooth and shiny to longitudinally costate or reticulate, inner area of dorsal surface of mandibles finely reticulate and opaque, propodeal lobe angulate, apex blunt. *Nesomyrmexasper* presents intraspecific morphological variation in some traits throughout its distribution in the Colombian TDF. The lateral tubercles of the petiole and postpetiole are variable characteristics, both in quantity and size, between populations of the species (Fig. 18). For example, workers from populations in the Valle del Cauca (western Colombia) have no lateral tubercles on the petiole and postpetiole (Fig. 18A) while in more northern populations such as those from La Guajira and Magdalena there are between one and three tubercles distributed asymmetrically on the sides of the petiole (Fig. 18D, E). Analysis of *N.asper* specimens across the Colombian TDF suggests that populations of this species are morphologically cohesive in terms of the diagnostic characteristics that define it, but traits such as lateral tubercles on the petiole/postpetiole show extensive phenotypic plasticity.

Our novel observations of variability in number and shape of lateral petiole and postpetiole tubercles in *N.asper* contrasts with the use of presence and absence of tubercles as a diagnostic trait for some nomenclatural changes in *N.asper* (Kempf 1958, 1959, 1975; Longino 2006). Longino (2006) proposed *Nesomyrmextristani* (Emery, 1896) as a junior synonym of *Nesomyrmexasper* considering the similarity of the lateral processes (i.e., lateral tubercles) of the petiole between the workers of both taxa. The same author used the differentiation of the lateral processes of the postpetiole (described there as “acuminate teeth”: p. 136) observed between the workers of Nesomyrmexaspervar.antoniensis (Forel, 1912) and *N.asper* s. str, to recognize *N.antoniensis* (Forel, 1912) as a valid species. Considering the intraspecific morphological variation in the lateral tubercles of *N.asper* workers, we compared this trait with the type worker of *N.antoniensis* (CASENT0909002), finding that the latter falls within the high intraspecific variability of *N.asper*. Likewise, the type specimens of both *N.antoniensis* (CASENT0909002) and *N.asper* (CASENT0901801), and the workers of *N.asper* present in Colombia and the Sierra Nevada de Santa Marta (type locality of *N.antoniensis*) were measured, finding that the type of *N.antoniensis* (HL 0.89, HW 0.83, SL 0.72, ML 0.43) matches the morphometric range of *N.asper* (HL 0.86–1.08, HW 0.78–0.99, SL 0.68–0.84, ML 0.41–0.53). Based on the evidence, we found that the morphological variation between *N.antoniensis* and *N.asper* is not distinct, with the form referred to as *N.antoniensis* falling within the variability observed in *N.asper*. Therefore, we propose *N.antoniensis* as a junior synonym of *Nesomyrmexasper*. The highly variable number and shape of tubercles on lateral petiole and postpetiole in *N.asper* should not be used to separate taxonomic units in these species groups. Interestingly, the lateral processes of the petiole and postpetiolar node described as “acuminate teeth” by Longino (2006: 136) are setigerous tubercles (Fig. 19; also see Kempf 1959) and their variation in shape is due to projection and orientation relative to the body.

#### ﻿ Nesomyrmex echinatinodis

Taxon classificationAnimaliaHymenopteraFormicidae

(Forel, 1886)

CBF0E959-A34B-5127-9F9D-39F361F67CA8

Figs 21 , 29

Leptothorax echinatinodis Forel, 1886: 48. Holotype (worker): Brazil, Rio de Janeiro. [NHMW] (AntWeb image examined, CASENT0909004).
Combination in Leptothorax (Goniothorax): Emery 1896: 59.
Leptothorax (Goniothorax) aculeatinodis Emery, 1896: 60. Holotype (worker): Costa Rica, Jiménez. [MSNG]. (AntWeb image examined, CASENT090475). Synonymy by Kempf 1959: 425.
Leptothorax (Goniothorax) pungentinodis Emery, 1896: 2. Holotype (queen): Panama, Gulf of Darien. [MSNG]. (AntWeb image examined, CASENT0904726) Synonymy by Kempf 1959: 425.
Leptothorax echinatinodis r. dalmasi Forel, 1899: 55. Syntype (worker): Colombia, Sierra Nevada de Santa Marta, San Antonio [MHNG]. (AntWeb image examined, CASENT0909005). Synonymy by Kempf 1959: 425.
Combination in Leptothorax (Caulomyrma): Forel 1914: 233.
Leptothorax (Goniothorax) echinatinodis subsp. schmidti Menozzi, 1927: 275 Syntype series (male, queen, worker): Costa Rica, San José. [DEIB]. (AntWeb image examined, FOCOL0198-1). Synonymy by Kempf 1959: 426.
Leptothorax (Goniothorax) echinatinodis subsp. cordincola Wheeler, 1942: 205. Syntype (worker): Panama, Canal zone, Chivachiva Trail. [MCZC]. (AntWeb image examined, CASENT0912925). Synonymy by Kempf 1959: 426.
Combination in Leptothorax (Nesomyrmex): Smith 1950: 30.
Combination in Nesomyrmex: Bolton 2003: 272.

##### Worker measurements.

(*n* = 15) HL 0.55–0.7, HW 0.49–0.65, SL 0.38–0.54, ML 0.28–0.35, EL 0.11–0.19, PW 0.30–0.45, PTW 0. 15–0.26, PPW 0.19–0.31, WL 0.63–0.91, PH 0.17–0.28, PTL 0. 20–0.34, PTH 0.16–0.25, PPL 0.19–0.35, PPH 0.17–0.25, GL 0.67–0.93. CI 89–92, SI 77–83.

**Figure 21.**
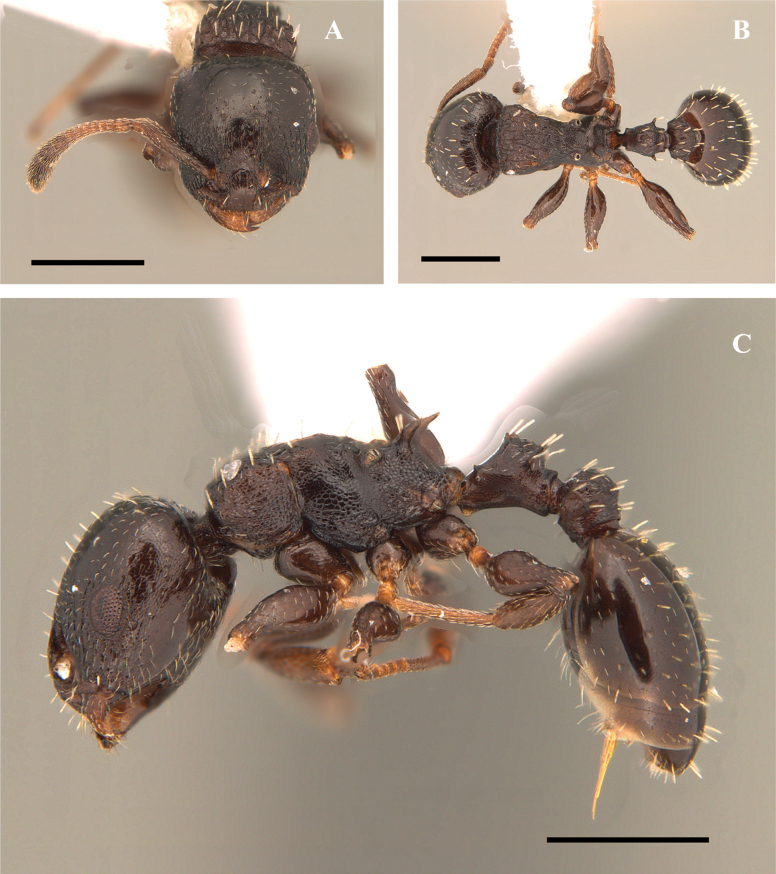
*Nesomyrmexechinatinodis* worker (MEFLG-NC 53153) **A** full-face view **B** lateral view **C** dorsal view. Scale bars: 0.5 mm.

##### Geographic range.

Argentina, Brazil, Colombia, Costa Rica, Ecuador, French Guiana, Guatemala, Guyana, Honduras, Mexico, Nicaragua, Panama, Paraguay, Peru, Suriname, Venezuela

##### Examined material.

Colombia • 1 worker; Antioquia, Santa Fe de Antioquia, Vda. El Espinal, Estación Agraria Cotové, Universidad Nacional de Colombia Sede Medellín; 6.5613889°N, 75.83166667°W; 600 m a.s.l.; 06 Oct. 2000; E. Vergara, F. Serna legs.; manual coll; UNAB. • 1 worker; Antioquia, Santa Fé de Antioquia, Vda. El Espinal, Estación Agraria Cotové, Universidad Nacional de Colombia Sede Medellín; 6.5358333°N, 75.82972222°W; 515 m a.s.l.; 02 Apr. 2020; B. Arredondo leg.; MEFLG-NC 53153. • 2 workers; Arauca, Tame, La Casirba; 6.360678°N, 71.894257°W; 675 m a.s.l.; 20 Mar. 2024; H. Sierra y S. De La Hoz legs.; Manual coll; CBUMAG:ENT:41658. • 1 worker; Arauca, Tame, La Casirba; 6.360678°N, 71.894257°W; 675 m a.s.l.; 19 -21 Mar. 2024; H. Sierra y S. De La Hoz legs.; Winkler; CBUMAG:ENT:41659. • 1 worker; Bolívar, San Jacinto, SFF los Colorados, La Yaya; 9.899947°N, 75.116645°W; 280 m a.s.l.; 06 -21 Apr. 2001; E. Deulefe leg.; Malaise trap; IAvH-E-251264. • 9 workers; Cundinamarca, Anapoima; 4.566113°N, 74.529935°W; 25 Mar. 1981; I. Zenner leg.; CTNI-NC 8245-1, 8245-2, 8245-3, 8245-4, 8245-5, 8245-6, 8245-7, 8245-8, 8245-9. • 1 worker; Cesar, Codazzi, Cgto. Casacará, Vda. Villa Matilde, RNSC Altahona-Castro; 9.793531°N, 73.190414°W; 403 m a.s.l.; 22 -24 Sep. 2023; H. Sierra, L. Ramos, R.J. Guerrero, S. De La Hoz legs.; Pitfall.; CBUMAG:ENT:41660. • 1 worker; La Guajira, Barranca, Cgto. San Pedro; 10.878276°N, 72.704619°W; 422 m a.s.l.; 10 -11 Sep. 2023; H. Sierra, S. De la Hoz, R.J. Guerrero, L. Ramos legs.; Manual coll.; CBUMAG:ENT:41662. • 1 worker; La Guajira, Barranca, Cgto. San Pedro; 10.876713°N, 72.704164°W; 404 m a.s.l.; 10 -11 Sep. 2023; H. Sierra, S. De la Hoz, R.J. Guerrero, L. Ramos legs.; Manual coll.; CBUMAG:ENT:41663. • 1 worker; La Guajira, Barranca, Cgto. San Pedro; 10.913786°N, 72.733971°W; 221 m a.s.l.; 09 -11 Sep. 2023; H. Sierra, L. Ramos, R. Guerrero, S. De La Hoz legs.; Winkler.; CBUMAG:ENT:41661. • 1 worker; Magdalena, Santa Marta, Parque Nacional Natural Tayrona, sector Zaino, sendero 9 Piedras; 11.2833333°N, 74.18333333°W; 600 m a.s.l.; C. Martínez leg.; manual coll.; ICN 106594. • 1 worker; Sucre, Ovejas; 9.53°N 75.23361111°W; 277 m. a.s.l.; 05 Apr. 2016; H. Cadena leg.; manual coll.; MEFLG-NC 48120. • 1 worker; Sucre, Ovejas; 9.5346111°N, 75.22086111°W; 277m. a.s.l.; 05 Apr. 2016; H. Cadena leg.; manual coll.; MEFLG-NC 48121. • 3 workers; Valle del Cauca, Santander de Quilichao, Las Chatas; 3.1096°N, 76.5265°W; 909 m a.s.l.; 17 Aug. 2021; M. A. Bautista-Giraldo; I. Armbrecht legs.; meadow; CBUMAG:ENT:40123, CBUMAG:ENT:40124, CBUMAG:ENT:40125.

##### Natural history.

Workers were collected in dry forest fragments associated with mango (*Mangiferaindica* L.) and Uvito (*Cordiadentata* Poir.) trees. Several workers were collected foraging on the ground on secondary TDF fragments in Arauca. A single record comes from the Malaise trap inside the mature TDF in SFF Los Colorados, to the northwest of Colombia. These ants are distributed from sea level up to 909m altitude.

##### Comments.

*Nesomyrmexechinatinodis* populations throughout its distribution in Colombia (unpublished data), including those from TDF, show wide morphological variability as noted by Kempf (1959) in his discussion of this species. For example, the sculpture on the head and mesosoma may present intra and intercolonial variability, but all the ants studied here share the diagnostic traits proposed by Kempf (1959) for this species, i.e., 11 antennomeres, mesosoma at least partly dark-colored, head with frontal and vertex area partially smooth and shiny, clypeus smooth and shiny, propodeal lobe short and uniformly rounded, basal third of the first gastral tergum slightly striate, were determined as *N.echinatinodis* s. str.

*Nesomyrmexechinatinodis*, *N.spininodis*, and *N.konina* sp. nov. share several traits, thus form the *echinatinodis* group, which can be recognized by short scapes which in repose do not reach the occipital margin, laterally serrated mesonotum, long and curved propodeal spines, propodeal lobe short and uniformly rounded., petiolar and postpetiolar node with lateral tubercles, scapes and legs lacking erect hairs.

*Nesomyrmexechinatinodis* has been previously recorded for the departments of Cundinamarca and La Guajira (Kempf 1959), Huila (Fernández et al. 1996), Magdalena (Fernández and Serna 2019), and Valle del Cauca (Armbrecht et al. 2001, Chacón de Ulloa et al. 2012). *Nesomyrmexechinatinodis* is one of two species previously recorded for the tropical dry forest in Colombia (Valle del Cauca), extending its distribution to TDF fragments in the departments of Antioquia, Bolívar, Cundinamarca, and Sucre.

#### ﻿ Nesomyrmex iku sp. nov.

Taxon classificationAnimaliaHymenopteraFormicidae

6573CDD7-1D42-5FF0-AF74-C1A06F70EF7C

https://zoobank.org/44B9782F-3DBC-467D-A3F8-58CFB3F6A0E4

Figs 22 , 29

##### Type material.

***Holotype***. Colombia • 1 worker; Magdalena, Santa Marta, Sierra Nevada de Santa Marta; 10.816667°N, 73.650000°W; IAvH-E-75014 [IAvH]. ***Paratype*** (*n* = 1). 1 worker; same data as holotype; IAvH-E-75007 (CBUMAG).

**Figure 22.**
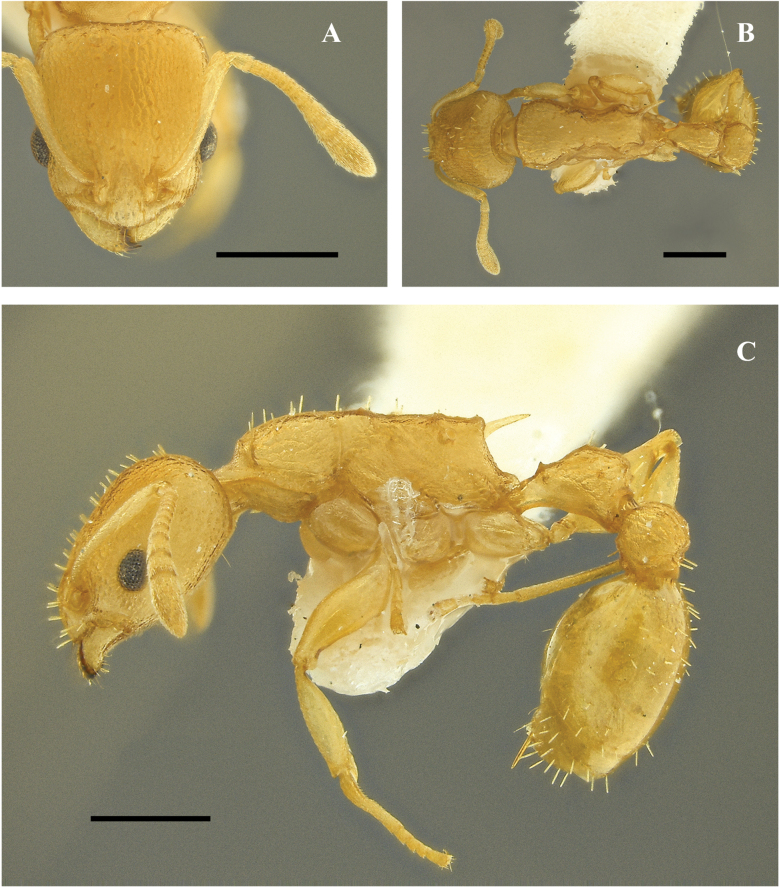
*Nesomyrmexiku* sp. nov. Holotype worker (IAvH-E-75014) **A** full-face view **B** lateral view **C** dorsal view. Scale bars: 0.5 mm.

##### Geographic range.

Colombia.

##### Holotype worker measurements.

HL 0.86, HW 0.73, SL 0.63, ML 0.34, EL 0.18, PW 0.48, PTW 0.25, PPW 0.28, WL 1.13, PH 0.34, PTL 0.47, PTH 0.30, PPL 0.3, PPH 0.29, GL 0.91. CI 85, SI 86.

##### Paratype worker measurements.

(*n* = 1) HL 0.89, HW 0.74, SL 0.63, ML 0.35, EL 0.18, PW 0.48, PTW 0.26, PPW 0.30, WL 1.18, PH 0.35, PTL 0.48, PTH 0.30, PPL 0.32, PPH 0.29, GL 0.96. CI 85, SI 86.

##### Diagnosis.

Mesosomal dorsum straight. Lateral margins of pronotum rounded, converging towards mesonotum. Lateral margins of mesonotum and dorsopropodeum with slightly angled lateral projections. Lateral dorsopropodeal processes short, not covering spiracle in dorsal view.

##### Description.

**Worker**. In full-face view, head longer than wide (CI 84–85), lateral margins straight, slightly curved posterior to the eyes, continuing into occipital margin, weakly convex mesally; mandible triangular, masticatory margin of mandible with five teeth, separated from basal margin by fifth tooth; anterior clypeal margin convex, projecting above the mandibles; lateral eyes protruding, below half the length of the head, with 11 ommatidia at their greatest diameter; full-face view, frontal lobes not prominent and weakly rounded, partially covering antennal insertions; frontal carina extending to anterior margin of eye; antenna with 12 antennomeres, 3-segmented club; scape curved at base, relatively long (SI 85–86) but not reaching the occipital margin by a distance almost twice its apical width; pedicel longer than wide, almost as long as the next three antennomeres together.

***Mesosoma*.** In lateral view, mesosomal profile straight; in dorsal view, pronotum wider than long, maximum width towards anterior margin, the latter slightly convex; humeral angles angulate; lateral margins of pronotum curved posteriorly; in lateral view, promesonotal suture marked; in dorsal view, lateral mesonotal and dorsopropodeal projections slightly angulated; propodeal spines divergent, longer than half the distance between their apices; propodeal spiracle small, circular, projecting posterodorsally; propodeal lobe short and uniformly rounded.

***Metasoma*.** In dorsal view, petiole trapezoidal with anterior portion narrower than posterior portion, lateral margins diverging posteriorly; in lateral view, peduncle and petiole node without apparent differentiation, forming a continuous outline, extending to dorsal face of petiole node; anterior face of petiole with anteromedial petiolar spine above petiolar spiracle; dorsal surface weakly rounded and short, continuing with a straight posterior face; anteroventral process of petiole pyramidal; in dorsal view, postpetiole oval, slightly wider than long, in lateral view postpetiole globose, taller than long.

***Sculpture*.** Body generally opaque; body surface rugose-reticulate, excluding smooth, shiny gaster; clypeus with medial longitudinal costae; dorsal surface of mandible, scapes, and legs weakly punctate and shiny.

***Pilosity and color*.** Dorsal surface of body with flattened erect hairs, mostly separated by a distance equal to or greater than their length, hairs as long as base of propodeal spines; in full-face view, scapes partly covered with short decumbent hairs, no erect hairs present; legs devoid of erect hairs, with sparse simple decumbent hairs restricted to apex of femur; gaster with sparse erect hairs, separated from each other by a distance twice their length. Body concolorous yellow.

##### Queen and male.

Unknown.

##### Natural history.

No related information.

##### Etymology.

The name of this species refers to the Arhuaco people with ancestral lands close to the type locality of the species. The Arhuaco people self-identify as Iku, which translates as “people” in the Ika language (Tracy 1997). Iku (Arhuacos) - Guardians of life, defenders of the peaceful coexistence of men among themselves and with the forces of nature. This is a name in apposition and thus invariable.

##### Comments.

This species is only known from the type material. *Nesomyrmexiku* sp. nov. is easily recognized by the shape of the straight mesosomal profile and by the lateral dorsopropodeal processes short, not covering spiracle in dorsal view. Morphologically, the closest species is *Nesomyrmexxerophilus* sp. nov., but they can be differentiated mainly by the lateral projection of the frontal lobes, the latter being wider posterior to the torulus, whereas *N.iku* does not have such a projection on the frontal lobes. On the other hand, in *N.xerophilus* the propodeal spiracles are not visible in dorsal view, since the lateral portion of the dorsopropodeal projects above them, whereas in *N.iku* the propodeal spiracles are visible in dorsal view.

Another species with similar morphology is *N.brasiliensis*. When comparing the type workers of *N.iku* (IAvH-E-75014, IAvH-E-75007) with the type ones of *N.brasiliensis* (MZSP87370, MZSP87374) we found that both species share a straight mesosomal profile, petioles and postpetioles lacking tubercles and body concolorous yellow. However, they differ in several features: The antennal flagellum of *N.iku* has 12 antennomeres, while *N.brasiliensis* has 11; additionally, the sculpture of the frontal area in N.brasiliensis is foveated in contrast to the frontal area striated in *N.iku*. Other distinguishing characteristics include the length of the propodeal spines, and the size and shape of the antennal scapes. A more extensive discussion on the similarity of *N.brasiliensis*, *N.iku*, and *N.xerophilus* will be possible when more material can be analyzed.

#### ﻿ Nesomyrmex konina sp. nov.

Taxon classificationAnimaliaHymenopteraFormicidae

B5A74306-6B13-534E-B7B3-D9F3B0DC4E69

https://zoobank.org/BD83D862-28F6-41A7-B3D2-AB18EBB2371E

Figs 23 , 29

##### Type material.

***Holotype*.** Colombia • 1 worker; Arauca, Tame, Parcela del Humboldt; 6.359289°N, 71.894258°W; 675 m a.s.l.; 19 Mar. 2024–21 Mar. 2024; H. Sierra, S. de La Hoz legs.; Winkler; CBUMAG:ENT:35951 [CBUMAG]. ***Paratypes*** (*n* = 4). Colombia • 3 workers; same data as holotype; CBUMAG:ENT:40035, CBUMAG:ENT:40036, CBUMAG:ENT:41654 (CBUMAG). • 1 worker; Cundinamarca, Medina, Vda. Periquito; 4.512722°N, 73.426833°W; 1043 m a.s.l.; 07–21 Feb. 2019; MPUJ_ENT0064660 (MPUJ).

**Figure 23.**
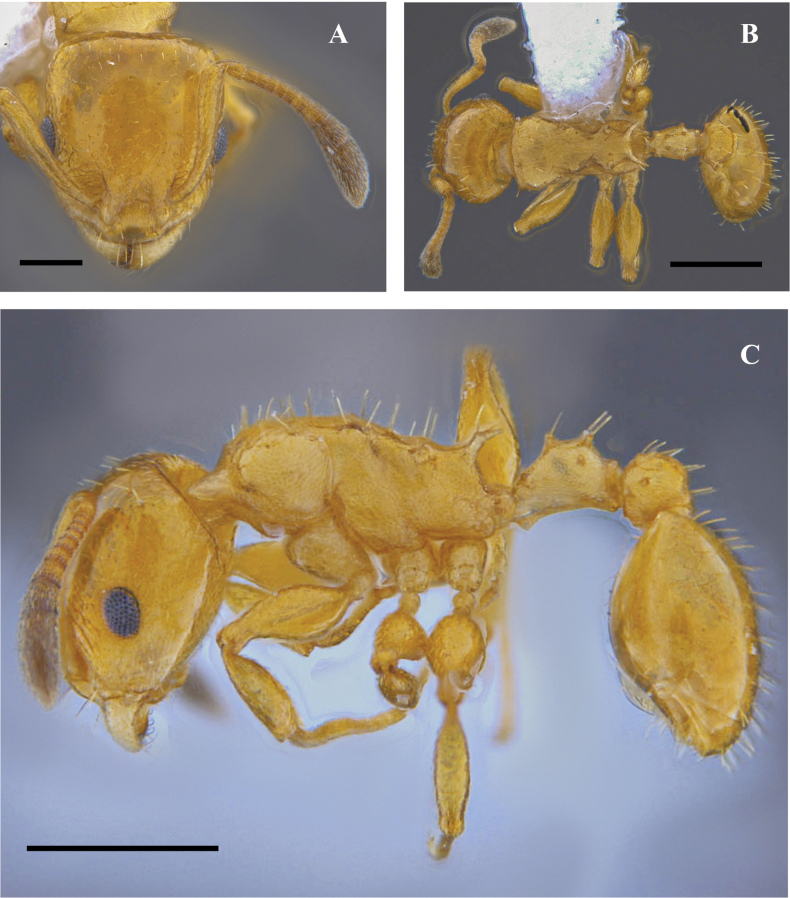
*Nesomyrmexkonina* sp. nov. Holotype worker (CBUMAG:ENT:35951) **A** full-face view **B** lateral view **C** dorsal view. Scale bars: 0.2 mm (**A**); 0.5 mm (**B, C**).

##### Holotype worker measurements.

HL 0.66. HW 0.57, SL 0.44, ML 0.26, EL 0.16, PW 0.4, PTW 0.19, PPW 0.25, WL 0.76, PH 0.24, PTL 0.23, PTH 0.2, PPL 0.21, PPH 0.21, GL 0.66. CI 86, SI 77.

##### Paratype worker measurements.

(*n* = 3). HL 0.66–0.67, HW 0.57–0.58, SL 0.44–0.46, ML 0.25–0.28, EL 0.15–016, PW 0.39–0.4, PTW 0.17–0.19, PPW 0.24– 0.25, WL 0.75–0.77, PH 0.21–0.26, PTL 0.21–0.24, PTH 0.19–0.2, PPL 0.18–0.21, PPH 0.20–0.21, GL 0.66–0.69. CI 86, SI 77–79.

##### Geographic range.

Colombia.

##### Diagnosis.

Dorsal surface of head and clypeus smooth and shiny. Clypeus without longitudinal median carina. Mesosome with slightly impressed longitudinal striation. Dorsal surface of mesosoma, petiole and postpetiole opaques. Legs and antennal scapes smooth and shiny. Basal third of first gastral tergum smooth and shiny.

##### Description.

**Worker.** In full-face view, head slightly longer than wide (CI 86), slightly narrow anterior to the eyes, posterolateral corners rounded, occipital margin weakly convex to straight; mandible triangular, inner mandibular margin with five teeth; anterior margin of clypeus weakly convex, projecting over mandibles; lateral eyes, with 10 ommatidia at greatest diameter, Posterior margin barely reaching midline of head length; frontal lobes straight, slightly expanded laterally, antennal insertions slightly exposed; front carina extending to anterior margin of eye; antenna of 11 antennomeres; scapes relatively short (SI 77), not extending past occipital margin, curved from base to mid-length; pedicel longer than wide, and nearly as long as next two antennomeres together; antennal club of three antennomeres.

***Mesosoma*.** In lateral view, mesosomal profile convex; in dorsal view, pronotum broader than long, greater width medially, anterior margin of pronotum slightly convex, humeral angle slightly angulated; in lateral view, promesonotal suture present, absent dorsally; lateral mesonotal projection rounded; lateral dorsopropodeal processes long, covering propodeal spiracle in dorsal; propodeal spines sharped divergent, as long as half the distance between their apices; in lateral view, propodeal spines forming an acute angle; propodeal spiracle small, circular, projecting posterodorsally; propodeal lobe short and uniformly rounded.

***Metasoma*.** In dorsal view, petiole subrectangular, anterior and posterior margins of petiole of equal width, lateral margins forming a rhombus, with their sides meeting towards the middle of their length; anterior and posterior margin of node with two tubercles of equal length located laterally; lateral margin of petiole node with a posterolateral tubercle; in lateral view, petiole trapezoidal, peduncle and petiole node without apparent differentiation, forming a continuous line, which extends to the dorsal face of the petiole node; anterior face of petiole with anteromedial petiolar spine on each side, above the petiole spiracle; dorsal face weakly rounded and short; anteroventral process of petiole developed, triangular in shape; in dorsal view, postpetiole hexagonal, twice as wide as long, with two lateral tubercles towards its middle length; in lateral view, postpetiole globose, taller than long.

***Sculpture*.** Dorsal surface of head mostly smooth and shiny, with reticulated area restricted to anterior area of eyes, continuing into frontal lobes and malar area; dorsal surface of mandibles partly smooth and shiny, with slight striation restricted to inner margin; clypeus smooth and shiny; scapes smooth and shiny; dorsum of mesosoma rugose-reticulate; lateral surface of mesosoma finely reticulate; petiole and postpetiole dull and rough-reticulate; gaster smooth and shiny.

***Pilosity and color*.** Dorsal surface of body, except head, with erect flattened hairs mostly separated by a distance equal to or greater than its length; hairs as long as base of propodeal spines; in full-face view, head capsule with simple, erect to suberect hairs; erect hairs restricted to vertex area; scapes covered with short decumbent hairs; propodeal spines with sparse erect hairs; legs with simple, appressed hairs, sparse and restricted to apex of femur; gaster with abundant erect hairs, separated by a distance equal to its length. Body yellowish brown, except for dark brown masticatory margin of mandible and antennomeres posterior to pedicel, varying from pale yellow to dark brown on antennal club.

##### Natural history.

Several of the type workers were extracted from the leaf litter in a fragment of tropical dry forest that has been recovering for more than 20 years in eastern Colombia.

##### Queen and male.

Unknown.

##### Etymology.

The word *konina* means smooth and shiny in the Sikuani aboriginal language, the language of the indigenous population that has shared the habitat where this species lives. The word is used to refer to the smooth and shiny sculpture of the cephalic dorsum of the ants of this species. This is a name in apposition and thus invariable.

##### Comments.

*Nesomyrmexkonina* is included in the group of echinatinodis species due to its morphological similarity (see comments in *N.echinatinodis*). It is distinguished from the other species in the group by its coloration and the smooth, shiny sculpture of the first gastral tergite.

#### ﻿ Nesomyrmex pittieri

Taxon classificationAnimaliaHymenopteraFormicidae

(Forel, 1899)

99D09D34-BB6C-5639-9A14-680FE250C1B0

Figs 24 , 29

Leptothorax pittieri Forel, 1899: 56. Holotype (worker): Costa Rica. [MHNG] (AntWeb image examined, CASENT0908997).
Combination in Leptothorax (Goniothorax): Emery 1924: 250.
Combination in Leptothorax (Nesomyrmex): Kempf 1958: 93.
Combination in Nesomyrmex: Bolton 2003: 272.

##### Worker measurements.

(*n* = 1) HL 0.81, HW 0.68, SL 0.53, ML 0.33, EL 0.21, PW 0.51, PTW 0.24, PPW 0.36, WL 0.98, PH 0.3, PTL 0.35, PTH 0.26, PPL 0.24, PPH 0.24, GL 0.78. CI 83, SI 78.

**Figure 24.**
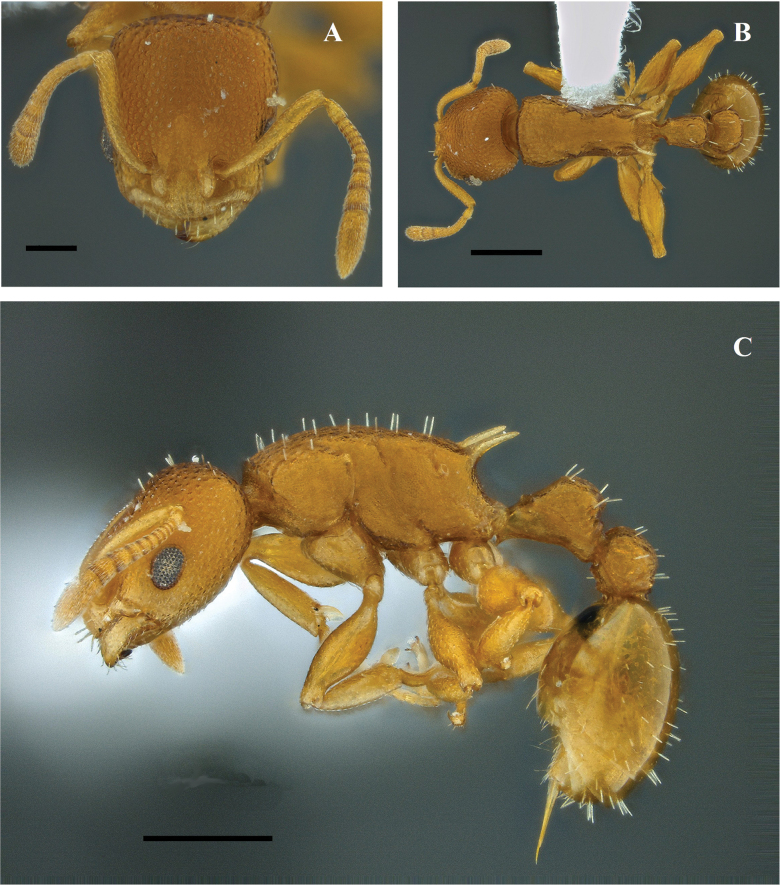
*Nesomyrmexpittieri* worker (CBUMAG:ENT:41664) **A** full-face view **B** lateral view **C** dorsal view. Scale bars: 0.2 mm (**A**); 0.5 mm (**B, C**).

##### Geographic range.

Colombia, Costa Rica, Guatemala, Honduras, Mexico, Nicaragua, Panama, Trinidad and Tobago.

##### Examined material.

Colombia • 1 worker; Valle del Cauca, Jamundí, Vda. San Isidro, Colindres; 3.33931°N, 76.5413°W; 979 m a.s.l.; 30 Jul. 2021; M.A. Bautista-Giraldo, I. Armbrecht legs.; CBUMAG:ENT:41664.

##### Natural history.

Species collected by manual sampling on vegetation in Parque Nacional Natural Tuparro.

##### Comments.

This species is recognized by the following characters: wide and deep foveae on the dorsal surface of the head, antennal scapes curved at their base, and propodeal spines as long as the distance between their tips.

The only specimen examined shows variation in coloration with respect to specimens from other forest types in Colombia (unpublished data). Populations found in premontane forest show bicoloration, with variations between reddish brown to dark. The sculpture of the frontal area is consistent in all populations (Fig. 24).

#### ﻿ Nesomyrmex pleuriticus

Taxon classificationAnimaliaHymenopteraFormicidae

(Kempf, 1959)

67EE9217-2485-54D4-9911-1ECCC135C492

Figs 25 , 29

Leptothorax (Nesomyrmex) pleuriticus Kempf, 1959: 414. Syntype series (male, queen, worker): Guyana, Kartabo. [MCZC, MZSP]. (image examined, MZSP87353, MZSP87354).
Combination in Nesomyrmex: Bolton 2003: 272.

##### Worker measurements.

(*n* = 1) HL 0.61, HW 0.58, SL 0.50, ML 0.25, EL 0.15, PW 0.36, PTW 0.16, PPW 0.22, WL 0.77, PH 0.21, PTL 0.242, PTH 0.16, PPL 0.18, PPH 0.19, GL 0.64.

**Figure 25.**
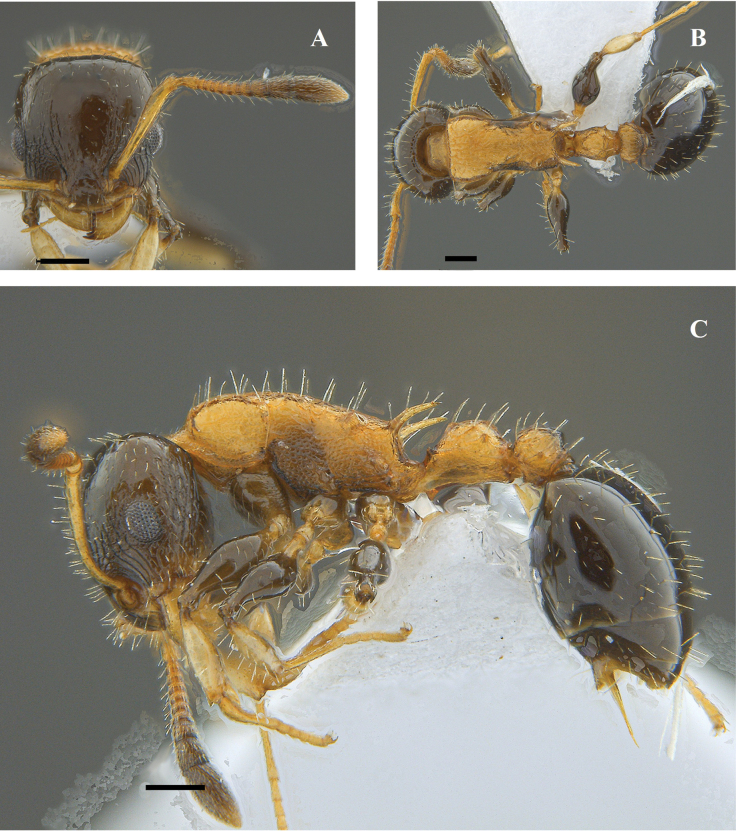
*Nesomyrmexpleuriticus* worker (IAvH-E-79866) **A** full-face view **B** lateral view **C** dorsal view. Scale bars: 0.2 mm.

##### Geographic range.

Bolivia, Brazil, Colombia, Costa Rica, Ecuador, French Guyana, Guatemala, Guyana, Honduras, Mexico, Panama, Surinam, Venezuela

##### Examined material.

Colombia • 1 worker; Vichada, Cumaribo, Cgto. Santa Rita, Parque Nacional Natural el Tuparro; 5.331667°N, 67.890833°W; 135 m a.s.l.; 08 Feb. 2004–10 Feb. 2004; I. Quintero; E. González legs.; Winkler; IAvH-E-79866.

##### Natural history.

Foragers were collected on vegetation.

##### Comments.

This species can be distinguished from other ones by having a straight anterior margin of the pronotum, an opaque mesosoma with striate sculpture and imbricate microsculpture, a dorsum of the postpetiole with longitudinal striations, scapes and legs covered with long, erect hairs, and a smooth, shiny clypeus.

The previous records of *N.pleuriticus* for the tropical dry forest in Colombia are based on information recorded for the departments of Cauca (Chacón de Ulloa et al. 2014) and Valle del Cauca (Armbrecht et al. 2001, Chacón de Ulloa et al. 2012) but we were unable to analyze these specimens to confirm the taxonomic identity of these ants (see comments on *N.vargasi*).

#### ﻿ Nesomyrmex spininodis

Taxon classificationAnimaliaHymenopteraFormicidae

(Mayr, 1887)

9409219A-903D-5F39-BBB8-2133FBBCAC1C

Figs 26 , 29

Leptothorax spininodis Mayr, 1887: 617. Lectotype worker: Brazil, Rio de Janeiro or Chile, Valparaiso. [NHMW]. (AntWeb image examined, CASENT0919734).
Combination in Leptothorax (Goniothorax): Emery 1896: 59.
Leptothorax (Goniothorax) umbratilis Wheeler, 1921: 160. Syntype (queen, worker): Guyana, Bartica, Penal Settlement. [MCZC, MZSP]. (Image examined, MZSP78366). Synonymy by Kempf 1959: 427.
Leptothorax (Goniothorax) genualia Santschi, 1922: 252. Holotype (worker): Paraguay, Asuncion. [NHMB]. (AntWeb image examined, CASENT0913001). Synonymy by Kempf 1959: 427.
Combination in Leptothorax (Nesomyrmex): Kempf 1959: 427
Combination in Nesomyrmex: Bolton 2003: 272.

##### Worker measurements.

(*n* = 1) HL 0.68, HW 0.62, SL 0.48, ML 0.29, EL 0.15, PW 0.44, PTW 0.21, PPW 0.28, WL 0.82, PH 0.25, PTL 0.32, PTH 0.22, PPL 0.22, PPH 0.22, GL 0.79, CI 91, SI 77.

**Figure 26.**
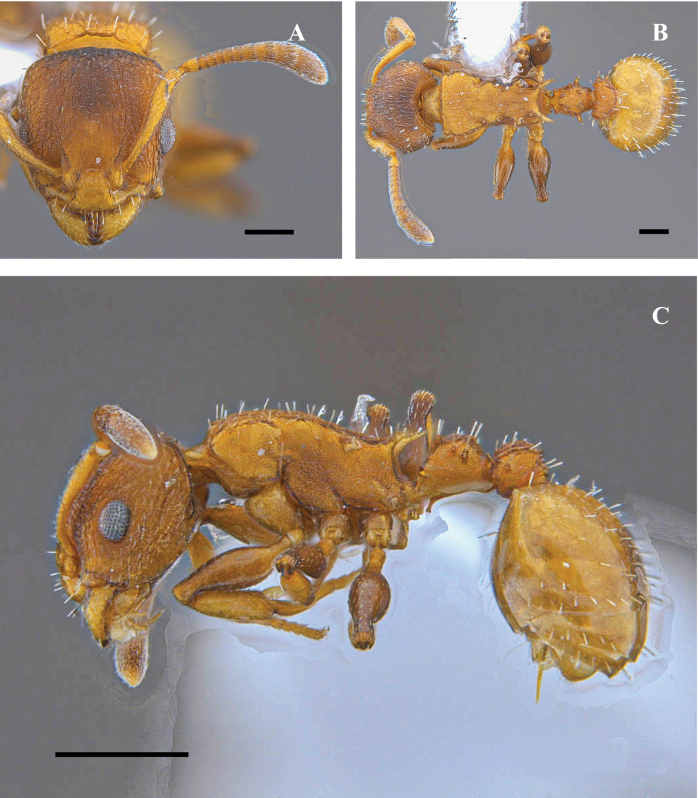
*Nesomyrmexspininodis* worker (IAvH-E-251254) **A** full-face view **B** lateral view **C** dorsal view. Scale bars: 0.2 mm (**A, B**); 0.5 mm (**C**).

##### Geographic range.

Argentina, Bolivia, Brazil, Colombia, Costa Rica, Ecuador, French Guiana, Paraguay, Suriname, Venezuela.

##### Examined material.

Colombia • 1 worker; Vichada, Cumaribo, Parque Nacional Natural Tuparro; 5.350278°N, 67.876667°W; 100 m a.s.l.; 12 Apr. 1995; J. Muñoz leg.; Manual coll.; IAvH-E-251254.

##### Natural history.

Species collected by manual sampling on vegetation in Parque Nacional Natural Tuparro.

##### Comments.

This species can be recognized by 11 antennomeres, uniformly testaceous or yellowish-brown coloration, dorsal surface of head finely reticulate-punctate and longitudinally rugose, without presence of shiny areas, and basal third of first gastral tergite generally aciculate-striate in its sculpture.

Kempf (1959) synonymized all previously described species and subspecies within the *echinatinodis* complex under the nominal species, except for *N.spininodis*. A potential revision of the *N.echinatinodis* complex could either result in the synonymization of certain species or the division of the complex into multiple forms. It is common to find *N.echinatinodis* exhibiting sculpture similar to that of *N.spininodis*, particularly in populations from Colombia different from those living in the TDF (Unpublished data). Additionally, coloration is highly variable within the *echinatinodis* complex. For this reason, using coloration as the primary criterion for distinguishing between different forms is not recommended. Thus, the separation of both species should not rely solely on coloration; instead, the sculpture of the frontal area of the head and the first gastral tergum should also be compared.

#### ﻿ Nesomyrmex vargasi

Taxon classificationAnimaliaHymenopteraFormicidae

Longino, 2006

50DBCF39-3F2D-54C5-83DC-84C22407947F

Figs 27 , 29

Nesomyrmex vargasi Longino, 2006: 136. Holotype worker: Costa Rica, Heredia Prov. [INBC]. (AntWeb image examined, JTLC000008517, JTLC000008518, LACMENT144699).

##### Worker measurements.

(*n* = 5) HL 0.70–0.76, HW 0.64–0.68, SL 0.54–0.61, ML 0.28–0.33, EL 0.15–0.16, PW 0.42–0.46, PTW 0.14–0.18, PPW 0.25–0.27, WL 0.88–0.96, PH 0.24–0.30, PTL 0.33–0.38, PTH 0.19–0.21, PPL 0.18–21, PPH 0.21–0.23, GL 0.79–0.91. CI 89–91, SI 84–89.

**Figure 27.**
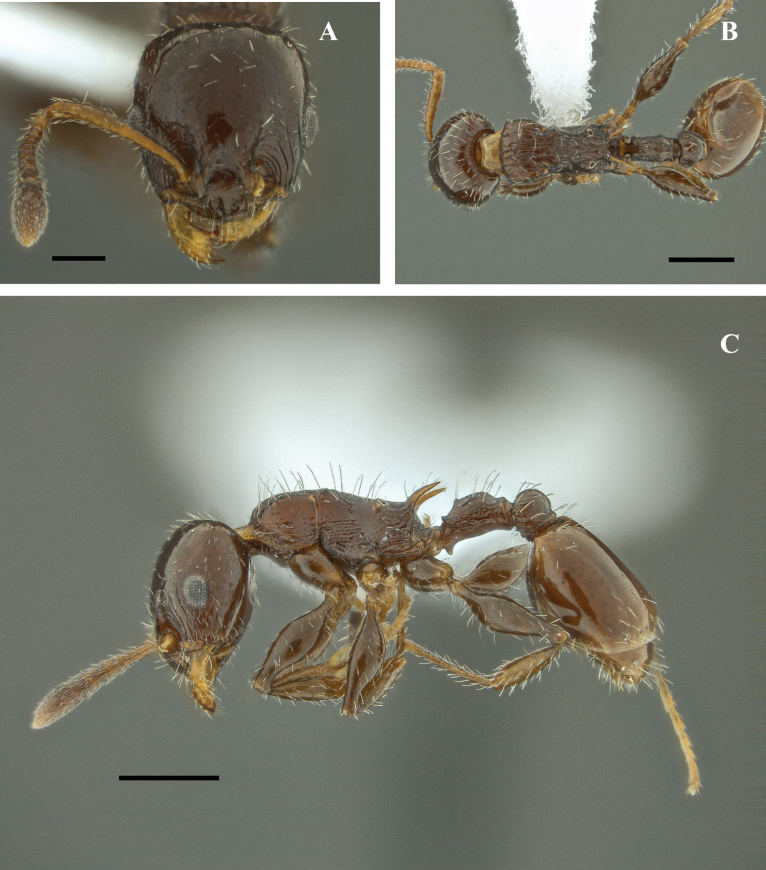
*Nesomyrmexvargasi* worker (CBUMAG:ENT:41665) **A** full-face view **B** lateral view **C** dorsal view. Scale bars: 0.5 mm.

##### Geographic range.

Colombia, Costa Rica.

##### Examined material.

Colombia • 1 worker; Bolivar, Zambrano; 9.744721°N, 74.82421°W; 1993–1994; Pitfall; IAvH-E-251258 • 1 worker; Cesar, Valledupar, Vda. Tierras nuevas; 10.241965°N, 73.761216°W; 881 m a.s.l.; 31 Mar. 2016; R. Achury leg.; Pitfall; CBUMAG:ENT:41665. • 1 worker; Vichada, Cumaribo, Cgto. Santa Rita, Parque Nacional Natural el Tuparro; 5.331667°N, 67.890833°W; 135 m a.s.l.; 08–10 Feb. 2004; I. Quintero; E. González legs.; Winkler; IAvH-E-79867.

##### Note.

Longino (2006) presents the diagnosis for *N.vargasi* but does not fully describe the species. To address the possible variability of this taxon, we offer a redescription of the worker caste based on specimens from Colombia.

##### Diagnosis.

Anterior margin of pronotum convex; mesosoma with marked longitudinal striations, space between the striae smooth and shiny; dorsum of postpetiole smooth and shiny; clypeus smooth and shiny; body surface smooth and shiny; antennal scape and legs with erect hairs (Longino, 2006).

##### Description.

**Worker.** In full-face view, head subquadrate, slightly longer than wide (CI: 89), slightly narrowing behind the eyes; occipital margin slightly straight; occipital angles rounded; lateral margins slightly convex, converging posteriorly; anterior margin of clypeus rounded, projecting over mandibles; mandible triangular; inner margin with five teeth, separated from basal margin by fifth tooth; lateral eyes, with 12 ommatidia at greatest diameter, situated toward mid-length of head; frontal lobes slightly expanded, antennal insertions partly exposed; front carina extending to anterior margin of eye; antenna with 11 antennomeres; scapes of moderate size (SI: 84), at rest reaching occipital margin; pedicel longer than wide, as long as next two antennomeres; antennal club with three antennomeres.

***Mesosoma*.** In lateral view, mesosomal profile convex; in dorsal view, pronotum wider than long, with its greatest width towards anterior margin, anterior margin of pronotum slightly convex; humeral angles slightly angulated; lateral margins of pronotum curved; in lateral view, pronotal suture marked; lateral dorsopropodeal processes (ldpp) long, covering propodeal spiracle in dorsal; propodeal spines of moderate size, slightly longer than the distance between their apices, in dorsal view propodeal spines diverge; in lateral view, propodeal spines curved; propodeal spiracle large, circular, projecting posterodorsally, diameter (0.071 mm) approximately equal to length of third and fourth antennomere; propodeal lobe angulate, apex blunt.

***Metasoma*.** In dorsal view, petiole rectangular, anterior and posterior margins of petiole of equal length; lateral margins of petiole parallel, twice the size of anterior margin; lateral margins of petiole node with 1 posterolateral tubercle on each side; in lateral view, petiole trapezoidal, peduncle and petiole node without apparent differentiation forming a continuous outline, extending to dorsal surface of petiole node; anterior surface of petiole with anteromedial petiolar spine on each side, superior to petiole spiracle; dorsal surface weakly rounded and short; anteroventral process of petiole broad, triangular in shape; in dorsal view, postpetiole cup-shaped, twice as wide as long; in lateral view postpetiole globose, taller than long.

***Sculpture*.** Dorsal surface of head mostly smooth and shiny, with a striate area restricted to the lower part of the frontal area, between the antennal insertions and the eyes; dorsal surface of mandibles smooth and shiny, with slight striation restricted to inner margin; clypeus smooth and shiny; scapes smooth and shiny; dorsum of mesosoma with longitudinal striations extending over entire surface; lateral surface of mesosoma smooth and shiny, with longitudinal striation restricted to lower margin; dorsum of petiole and postpetiole smooth and shiny; gaster smooth and shiny.

***Pilosity and color*.** Dorsal surface of body with erect hairs, separated by a distance less than the length; erect hairs as long as the length of propodeal spines; in full-face view, head capsule with simple, erect to suberect hairs; scapes covered with erect hairs as long as the maximum width of the scape; antennae and mandibles pale brown; propodeal spines and legs with erect hairs equal to the length of propodeal spines; gaster with abundant erect hairs, separated by a distance less than its length.

##### Natural history.

Ants were collected in lowlands, foraging on the ground and in leaf litter.

##### Comments.

According to Longino (2006), *N.vargasi* and *N.pleuriticus* are parapatric species differentiated by the sculpture of the body surface, much smoother and shinier in *N.vargasi*. The latter is distributed in humid forests at medium elevations of ~ 1100 m, while *N.pleuriticus* lives in lowlands, below 500 m. In Colombia, the populations of *N.vargasi* and *N.pleuriticus* present a sympatric distribution since workers of both species were collected as foragers on the vegetation in the same fragment of TDF in the Tuparro National Natural Park (Vichada). This record increases the distribution range of *N.vargasi* from 135 m in the tropical dry forest to 1100 m in humid forest.

The examined workers of *N.vargasi* fit the definition of Longino (2006), with a smooth and shiny body, although they are slightly paler in color. Sympatric populations of *N.pleuriticus* and *N.vargasi* show variation in size, sculpture, coloration and shape of the petiole and postpetiole, exhibiting contrasting characteristics between both species, reaffirming the description of *N.vargasi* by Longino (2006). This record expands the distribution of *N.vargasi*, being the first report for South America.

#### ﻿ Nesomyrmex xerophilus

Taxon classificationAnimaliaHymenopteraFormicidae

Arredondo-H & Guerrero sp. nov.

BC051E13-C821-5581-A984-0E21F9F60A48

https://zoobank.org/93376D41-ADB1-4AE0-AD39-E550F07516C7

Figs 28 , 29

##### Type material.

***Holotype*.** Colombia • 1 worker; Magdalena, Santa Marta, Vda. Puerto Mosquito, Reserva la Iguana Verde; 11.176972°N, 74.185167°W; 02 Nov. 2019; M. Escárraga leg.; Manual capture; CBUMAG:ENT:12440 [CBUMAG]. ***Paratypes*** (*n* = 14). Colombia • 8 workers; same data as holotype; CBUMAG:ENT:12441 (ICN), CBUMAG:ENT:41656 (CBUMAG), CBUMAG:ENT:42097 (CBUMAG), CBUMAG:ENT:42098 (CBUMAG), CBUMAG:ENT:42099 (CBUMAG), CBUMAG:ENT:42100 (CBUMAG), CBUMAG:ENT:42101 (CBUMAG), CBUMAG:ENT:42102 (MEFLG). • 1 worker; Cesar, Valledupar, Cgto. Los Corazones, PNR Los Besotes; 10.572165°N, 73.271218°W; 584 m a.s.l.; 14 16 Sep. 2023; H. Sierra, L. Ramos, R. Guerrero y S. De La Hoz legs.; Winkler; CBUMAG:ENT:41655 (CBUMAG). • 2 workers; Magdalena, Santa Marta, Reserva Caoba; 11.213139°N, 74.101667°W; 27 Nov. 2018; M. Escárraga leg.; CBUMAG:ENT:12164, CBUMAG:ENT:12165 (CBUMAG). • 1 worker; Magdalena, Santa Marta, Cerro Taganga; 11.259111°N, 74.180139°W; 117.3 m a.s.l.; 03 Mar. 2007; D. Ramírez y D. Olivero-G legs.; Pitfall; CBUMAG:ENT:12163 (IAvH). • 2 workers. Magdalena, Santa Marta, Ciénaga, 5 km SE, Río Frío; 10.883306°N, 74.133333W; 100 m a.s.l.; 16 ago. 1985; P. Ward leg.; ICN 019704 (ICN).

**Figure 28.**
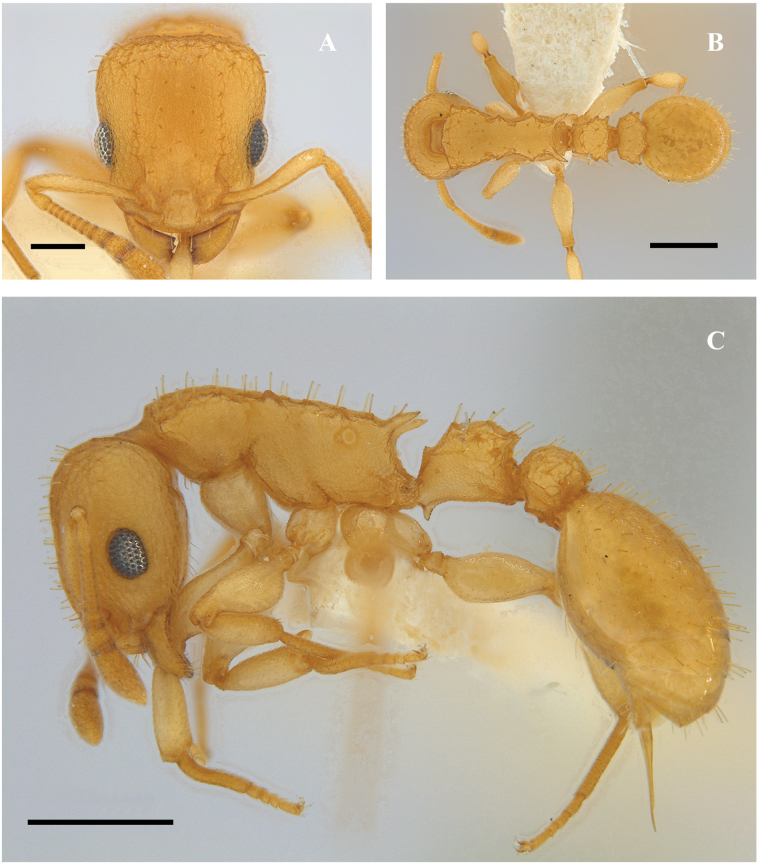
*Nesomyrmexxerophilus* sp. nov. Holotype worker (CBUMAG: ENT 12440) **A** full-face view **B** lateral view **C** dorsal view. Scale bars: 0.2 mm (**A**); 0.5 mm (**B, C**).

**Figure 29.**
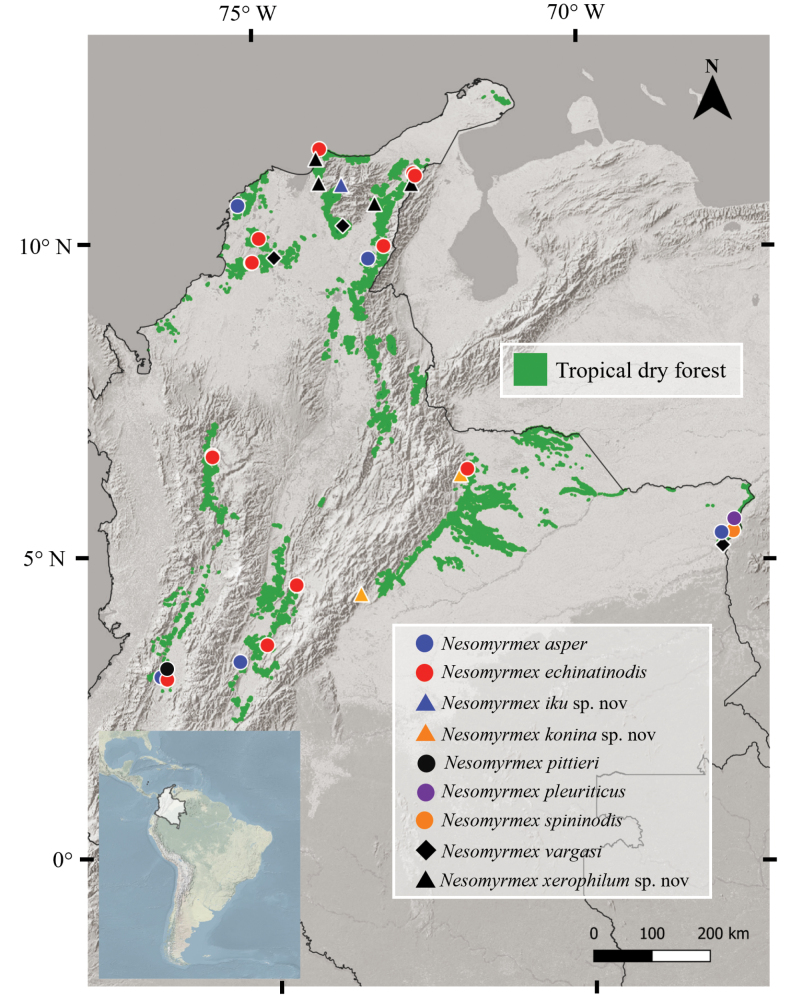
Distributional map of *Nesomyrmex* species from the tropical dry forest in Colombia.

##### Geographic range.

Colombia.

##### Holotype worker measurements.

HL 0.69, HW 0.6, SL 0.44, ML 0.29, EL 0.15, PW 0.43, PTW 0.28, PPW 0.37, WL 0.88, PH 0.23, PTL 0.23, PTH 0.25, PPL 0.22, PPH 0.23, GL 0.66 CI 86, SI 0.73.

##### Paratype worker measurements.

(*n* = 4) HL 0.69–0.7, HW 0.59–0.6, SL 0.43–0.44, ML 0.29, EL 0.15–0.16, PW 0.43–0.44, PTW 0.28–0.29, PPW 0.36–0.37, WL 0.9–0.91, PH 0.22–0.23, PTL 0.23–0.27, PTH 0.24–0.25, PPL 0.22–0.23, PPH 0.23–0.24, GL 0.66–0.68, CI 85–86, SI 72–73.

##### Diagnosis.

Frontal lobe projected laterally, covering the antennal insertions. Frontal carina extending to posterior cephalic vertex, forming a weak scrobus. Dorsum of mesosoma straight and flattened. Lateral margin of pronotum straight, converging towards mesonotum. Lateral projection of the mesonotum dentate. Lateral dorsopropodeal processes long, covering propodeal spiracle in dorsal view.

##### Description.

**Worker.** In full-face view, head longer than wide (CI 85–86), slightly narrowing behind the eyes; occipital margin weakly convex; occipital angles rounded; lateral margins slightly curved posteriorly; anterior margin of clypeus weakly convex, projecting over mandibles; eyes lateral, with 10 ommatidia at greatest diameter; frontal lobes laterally expanded, antennal insertions completely hidden by frontal lobes; frontal carina extending to posterior cephalic vertex, as if forming a weak scrobus; antenna with 12 antennomeres; mandible triangular; inner margin with five teeth, separated from basal margin by fifth tooth; scapes short (SI 72–73) barely reaching posterior margin of eye, curved towards mid-length; pedicel longer than wide, and almost as long as next three antennomeres together; antennal club with three antennomeres.

***Mesosoma*.** In lateral view, mesosomal profile straight; in dorsal view, pronotum wider than long, with its maximum width towards anterior margin, the latter slightly convex, humeral angle angulated, lateral margins of pronotum curved; in lateral view, promesonotal suture present, in dorsal view, absent; in dorsal view, mesosomal lateral projection angulate; dorsopropodeal lateral projection angulate, projecting over propodeal spiracles; propodeal spines short, less than half the distance between their apices in length, and divergent; in lateral view, propodeal spiracle small (diameter = 0.057 mm), approximately equal to the length of the 10^th^ antennomere, circular, projected posterolaterally; propodeal lobe short and uniformly rounded.

***Metasoma*.** In dorsal view, petiole trapezoidal, anterior margin shorter than posterior margin; posterior margin with two mesial tubercles of equal length; lateral margins diverging from anterior to posterior margin; lateral margin with two posterolaterals tubercles; in anteroposterior direction, first tubercle poorly developed, length less than half the total length of the following tubercle; in lateral view, petiole subquadrate, peduncle and petiolar node without apparent differentiation, forming a continuous outline, which extends to the dorsal surface of the petiolar node; anterior surface of petiole with anteromedial petiolar spine on each side, superior to the petiolar spiracle; dorsal surface weakly rounded and short; anteroventral process of petiole acute; in dorsal view, postpetiole hexagonal, twice as wide as long; in lateral view postpetiole globose, taller than long.

***Sculpture*.** Body generally opaque, with smooth, shiny areas restricted to gaster; dorsal surface of body rugose-reticulate, excluding gaster; lateral surface of mesosoma reticulate; clypeus smooth and shiny, with medial longitudinal carina; dorsal surface of mandibles shiny, with weakly marked striation; scape and legs shiny with superficial sculpture; gaster weakly imbricate.

***Pilosity and color*.** Dorsal surface of body with flattened erect hairs, mostly separated by a distance equal to or greater than its length; hairs as long as the base of the propodeal spines; in full-face view, head capsule with erect hairs restricted to the clypeus, area between the frontal carinae and vertex; scapes covered with short decumbent hairs, without erect hairs; propodeal spines with erect hairs; legs with simple, appressed, sparse hairs restricted to the apex of the femora; gaster with abundant erect hairs, separated by a distance equal to its length. Body pale yellow, legs slightly paler, base and apex of femur and tibia darker.

**Queen and male.** Unknown.

##### Natural history.

This species lives within dead branches of shrub vegetation. Populations are distributed in fragments ~ 100 m altitude. In all cases, the ants were found within dry forest with dense vegetation.

##### Etymology.

The epithet *xerophilus* is a word composed of the prefix *xero*- (from ancient Greek *ξηρο*- meaning dry) and the suffix -*philo* or -*philus* (from ancient Greek *φίλος* meaning attraction towards). The species epithet refers to the dry habitat where these ants live, that is, the tropical dry forest, a life zone highly threatened by the high rate of deforestation in Colombia. It is a noun in apposition and thus invariant.

##### Comments.

Species easily recognizable by the lateral expansion of the frontal lobes and the lateral projection of the mesosoma and dorsopropodeum in the form of an angled lobe. Morphologically, the closest species to *N.xerophilus* is *N.iku* (see comments on *Nesomyrmexiku* sp. nov.). *N.xerophilus* is morphologically similar to *N.wilda*, with both species exhibiting a lateral projection of the mesonotum dentate, long lateral dorsopropodeal processes covering of the propodeal spiracle in dorsal view. Additionally, they share the shape of the propodeal lobes, coloration, and sculpture. Despite these similarities, *N.xerophilus* differs from *N.wilda* in the number of antennomeres, as well as in the shape of the frontal lobes, petiole, and postpetiole.

Unlike *N.asper*, the position and number of petiolar and postpetiolar tubercles in *N.xerophilus* remain consistent across its populations, with no variation observed in this characteristic. This stability may be attributed to its limited distribution in the tropical dry forest of the Colombian Caribbean region. In contrast, the variation in the position and number of petiolar and postpetiolar tubercles in *N.asper* appears to be linked to its altitudinal distribution and geographic location (see comments on *N.asper*).

## ﻿General comments

The taxonomy of neotropical *Nesomyrmex* has been neglected, which has substantially limited the understanding of the morphological limits of the species and, therefore, their diversity, natural history, and distribution. This work is not a taxonomic revision of the genus *Nesomyrmex*, but it does seek to lay the foundations for future taxonomic approaches based on morphological characters aimed at solving nomenclatural problems and descriptions of new species within this genus. In this sense, we evaluate the taxonomic utility of the morphological traits used by Kempf (1959) and propose new characters for the delimitation of the species. Kempf (1959) used the presence or absence of petiole tubercles to argue the taxonomic limits of some species (e.g., *Nesomyrmexasper*), however, the analysis of that characteristic throughout the distribution of *N.asper* in the Colombian dry forest shows that petiolar tubercles can be phenotypically variable. In contrast to the variability in petiolar tubercles, we were able to determine that the sculpture of the dorsum of the clypeus together with the shape of the propodeal lobe are useful traits in the identification of this species. Body surface sculpture, mainly on the head, was also widely used by Kempf (1959) to define taxonomic boundaries of some species; for example, that same author defined *Nesomyrmexechinatinodis* as having head with frontal and vertex area partially smooth and shiny, however, when exploring in detail the head sculpture in workers of that species variability is observed, since the sculpture on the head of *N.echinatinodis* can range from smooth and shiny, finely reticulate-punctate to longitudinally rugose, the latter similar to that found in *Nesomyrmexspininodis*. This phenotypic convergence between some workers of *N.echinatinodis* and the workers of *N.spininodis* can generate confusion in the identification between both species, however, the sculpture in the first gastral tergite separates them from each other (see comments on *N.spininodis*).

Although head sculpture generated uncertainty in the identification between a pair of species, body sculpture was consistent and useful for identifying between *Nesomyrmexpleuriticus* and *Nesomyrmexvargasi* in the Colombian TDF. In both dry forest and other regions of Colombia (unpublished data), *Nesomyrmexpleuriticus* workers have anastomosing longitudinal striations on the dorsum of the mesosoma and marked reticule-punctate sculpture on the mesopleura, in broad contrast to the parallel longitudinal striations without microsculpture (smooth, shiny surface appearance) in *N.vargasi*. The sculpture of the dorsum of both the mandible and the clypeus, the shape and expansion of the frontal lobes, the length of the scape, as well as the sculpture of the first gastral tergum were shown to be taxonomically useful and should be used for a broader taxonomic approach.

Six ant species have been recorded in Colombia (Fernández and Serna 2019). Our study, conducted in a little-explored ecosystem, adds three new species and one new record, expands the known distribution of others, and includes a morphological analysis. Based on these findings, we propose nomenclatural changes, suggest new species, and provide additional biological data. Also, we identified the regions that concentrate the highest number of *Nesomyrmex* species in their TDF fragments. Both the Caribbean and the Eastern regions have five species of the nine recorded here. Interestingly, new species are recorded from each of these regions, two for the Caribbean region and one for the Eastern region. Likewise, our results show that *N.asper* and *N.echinatinodis* are the most widely distributed species in the Colombian TDF.

## Supplementary Material

XML Treatment for Nesomyrmex asper

XML Treatment for Nesomyrmex echinatinodis

XML Treatment for Nesomyrmex iku

XML Treatment for Nesomyrmex konina

XML Treatment for Nesomyrmex pittieri

XML Treatment for Nesomyrmex pleuriticus

XML Treatment for Nesomyrmex spininodis

XML Treatment for Nesomyrmex vargasi

XML Treatment for Nesomyrmex xerophilus

## ﻿Additional information

### Author ORCIDs

Brandon S. Arredondo https://orcid.org/0000-0003-2777-5909

Roberto J. Guerrero https://orcid.org/0000-0003-3244-2754

### Data availability

All of the data that support the findings of this study are available in the main text.
